# Production of *p*-amino-l-phenylalanine (l-PAPA) from glycerol by metabolic grafting of *Escherichia coli*

**DOI:** 10.1186/s12934-018-0996-6

**Published:** 2018-09-21

**Authors:** Behrouz Mohammadi Nargesi, Natalie Trachtmann, Georg A. Sprenger, Jung-Won Youn

**Affiliations:** Institute of Microbiology, University of Stuttgart, Allmandring 31, 70569 Stuttgart, Germany

**Keywords:** *Escherichia coli*, Non-proteinogenic aromatic amino acids, *p*-Amino-l-phenylalanine, Metabolic grafting

## Abstract

**Background:**

The non-proteinogenic aromatic amino acid, *p*-amino-l-phenylalanine (l-PAPA) is a high-value product with a broad field of applications. In nature, l-PAPA occurs as an intermediate of the chloramphenicol biosynthesis pathway in *Streptomyces venezuelae*. Here we demonstrate that the model organism *Escherichia coli* can be transformed with metabolic grafting approaches to result in an improved l-PAPA producing strain.

**Results:**

*Escherichia coli* K-12 cells were genetically engineered for the production of l-PAPA from glycerol as main carbon source. To do so, genes for a 4-amino-4-deoxychorismate synthase (*pabAB* from *Corynebacterium glutamicum*), and genes encoding a 4-amino-4-deoxychorismate mutase and a 4-amino-4-deoxyprephenate dehydrogenase (*papB* and *papC*, both from *Streptomyces venezuelae*) were cloned and expressed in *E. coli* W3110 (lab strain LJ110). In shake flask cultures with minimal medium this led to the formation of ca. 43 ± 2 mg l^−1^ of l-PAPA from 5 g l^−1^ glycerol. By expression of additional chromosomal copies of the *tktA* and *glpX* genes, and of plasmid-borne *aroFBL* genes in a *tyrR* deletion strain, an improved l-PAPA producer was obtained which gave a titer of 5.47 ± 0.4 g l^−1^ l-PAPA from 33.3 g l^−1^ glycerol (0.16 g l-PAPA/g of glycerol) in fed-batch cultivation (shake flasks). Finally, in a fed-batch fermenter cultivation, a titer of 16.7 g l^−1^ l-PAPA was obtained which is the highest so far reported value for this non-proteinogenic amino acid.

**Conclusion:**

Here we show that *E. coli* is a suitable chassis strain for l-PAPA production. Modifying the flux to the product and improved supply of precursor, by additional gene copies of *glpX*, *tkt* and *aroFBL* together with the deletion of the *tyrR* gene, increased the yield and titer.

**Electronic supplementary material:**

The online version of this article (10.1186/s12934-018-0996-6) contains supplementary material, which is available to authorized users.

## Background

The general aromatic biosynthesis pathway (also called shikimate pathway) in most microorganisms and plants starts by condensation of the precursors phosphoenolpyruvate (PEP) and erythrose-4-phosphate (E4P) to provide the first intermediate, 3-deoxy-d-arabino-heptulosonate-7-phosphate (DAHP). Eventually (after introduction of another PEP molecule), the last common metabolite, chorismate [1] is formed. From chorismate, various biosynthetic pathways diverge which give rise to a plethora of aromatic compounds. Among them are three proteinogenic aromatic amino acids [l-phenylalanine (l-Phe), l-tyrosine (l-Tyr), and l-tryptophan (l-Trp)] and aromatic vitamins such as quinones, folate (via *p*-aminobenzoate), and *p*-hydroxybenzoate [2–5]. Quinones (ubiquinone, menaquinone, plastoquinone) fulfill important roles in the electron transport chain [3, 6]. Enterobactin (from 2,3-dihydroxybenzoate) is used as an iron complexing siderophore in *E. coli*, folate is involved in C1 metabolism, and vitamin E in photosynthetic organisms is involved in protection against oxidative stress [7–9]. Various secondary metabolites such as phenylpropanoids (mainly in plants) and some antibiotics (chloramphenicol, pristinamycin, and others) in streptomycetes are as well derived from the aromatic pathway [10–12].

Aromatic amino acids are currently produced in large scale by fermentations or biotransformations with genetically modified microorganisms like *E. coli* or *Corynebacterium glutamicum* [3–5, 7, 13–18]. Aromatic compounds find many applications in biotechnology, nutrition, and medicine. l-Phe is mainly used for the synthesis of the low-calorie sweetener aspartame (l-aspartyl- l-phenylalanine methyl ester) while l-Trp is used for pharmaceuticals and as animal feed [3, 16]. l-Tyr is used as dietary supplement in the treatment of phenylketonuria. It is also the precursor in the synthesis of Levodopa (l-3,4-dihydroxyphenylalanine) which is used to treat Parkinson’s disease [19]. Ubiquinone-10 (Q_10_), folic acid, and vitamin K (plastoquinone) are used as nutritional additives [20, 21].

The rare, non-proteinogenic aromatic amino acid, *para*-amino-l-phenylalanine (l-PAPA) is used for technical and pharmaceutical applications [22–25] and has been described as a building block of the anticancer drug, Melphalan^®^ [26, 27]. In nature, l-PAPA occurs in plant seeds (*Vigna vexillata*; [28]), and is an intermediate of the chloramphenicol and pristinamycin biosynthesis pathways in *Streptomyces venezuelae* and *S. pristinaespiralis* [29–31]. l-PAPA is also a precursor of the antibiotic obafluorin ß-lactone of *Pseudomonas fluorescens* [32] and of GameXPeptides in the entomopathogenic bacterium, *Photorhabdus luminescens* [33]. l-PAPA was moreover successfully used as precursor in the biosynthetic diversification of jadomycin production with *S. venezuelae* cells [34]. L-PAPA has also been used for the synthesis of the biopolyamide precursor, 4-aminohydrocinnamic acid [35, 36].

l-PAPA synthesis starts from chorismate which is first converted by a glutamine- or ammonia-dependent 4-amino-4-deoxychorismate synthase to give 4-amino-4-deoxychorismate (ADC) [37]. This step is common to both, the *para*-aminobenzoate (PABA) and the chloramphenicol/pristinamycin biosynthesis pathways. In PABA biosynthesis, ADC synthase is encoded by genes *pabA* and *pabB*—which are not organized in an operon—in *E. coli* [38]. In Corynebacteria, these genes are fused as *pabAB* such as in *C. glutamicum* [39–41], in *C. efficiens* [42], or *C. callunae* [40]. In pristinamycin biosynthesis, the pathway-specific gene is named *papA* in *S. pristinaespiralis* [30], and in chloramphenicol biosynthesis of *S. venezuelae* it is either termed *papA* [43] or *cmlB* [44], respectively. Despite the different nomenclature, these genes display high sequence identities [30, 43–45]. ADC is converted by 4-amino-4-deoxy-chorismate mutase (gene *papB* in *S. pristinaespiralis;* alternatively *cmlD* in *S. venezuelae*) and 4-amino-4-deoxyprephenate dehydrogenase (*papC* and *cmlC*, respectively) to yield *p*-aminophenylpyruvate (PAPP; [30, 43, 44]). By transamination, l-PAPA is formed and serves then as intermediate for the production of several antibiotics. Recently, *papA*,*B*,*C* homolog genes from *Pseudomonas fluorescens* have been successfully cloned and expressed in *E. coli* [24].

**Fig. 1:**
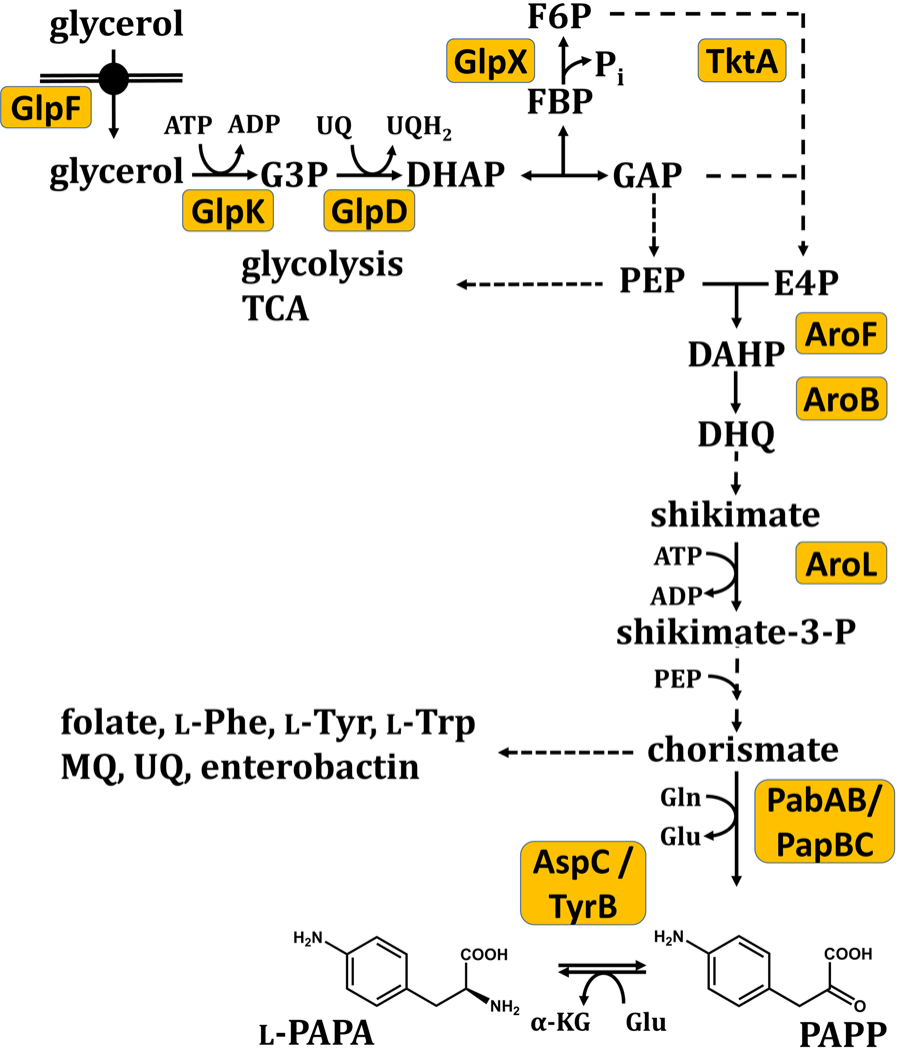
Overview of the *de novo* l-PAPA biosynthesis pathway from glycerol in *E. coli*. The scheme of reactions is modified from Gottlieb et al. [15]. Broken arrows indicate incomplete presentation of the metabolic pathway. *AroF* DAHP synthase, *AroB* dehydroquinate synthase, *AroL* shikimate kinase, *AspC* aspartate transaminase, *GlpD* glycerol-3-phosphate dehydrogenase, *GlpF* glycerol facilitator, *GlpK* glycerol kinase, *GlpX* fructose-1,6-bisphosphate phosphatase, *PabAB* 4-amino-4-deoxychorismate synthase, *PapB* 4-amino-4-deoxychorismate mutase, *PapC* 4-amino-4-deoxyprephenate dehydrogenase, *TktA* transketolase A, *TyrB* aromatic aminotransferase are presented

*Escherichia coli* is not a natural producer of l-PAPA but the *E. coli* aminotransferases TyrB and AspC are known to convert PAPP into l-PAPA (Fig. 1) [46, 47]. Earlier, l-PAPA formation in recombinant *E. coli* (carrying *papABC* genes from *S. venezuelae* on a low-copy plasmid) had been shown by the group of Schultz [43]. They used this in vivo produced compound as “21st proteinogenic amino acid” together with a modified tyrosyl-tRNA synthetase and a mutant tyrosine amber suppressor tRNA in specifically engineered *E. coli* strains. While formation of up to 0.7 mM of intracellular l-PAPA were measured in these recombinant cells, no data on extracellular production were provided [43], however.

A preferred carbon source for the production of aromatic amino acids with *E. coli* is glucose. Glycerol which can be used as an alternative carbon and energy source by *E. coli*, is especially attractive for the production of aromatic amino acids [15, 18, 48]. Glycerol can be taken up in *E. coli* either via the glycerol facilitator, GlpF or by unassisted diffusion [49]. After phosphorylation by the ATP-dependent glycerol kinase (GlpK; [50]), and an oxidation step by glycerol 3-phosphate dehydrogenase (GlpD; [51]) dihydroxyacetone phosphate (DHAP) can enter the lower glycolysis. Glycerol has no competing application in the food industry and it appears as a byproduct during biodiesel production [52]. Here we describe the construction of *E. coli* strains for the production of valuable l-PAPA using glycerol as sole carbon and energy source.

## Results

### Construction of a de novo l-PAPA biosynthesis pathway in *E. coli*

**Fig. 2:**
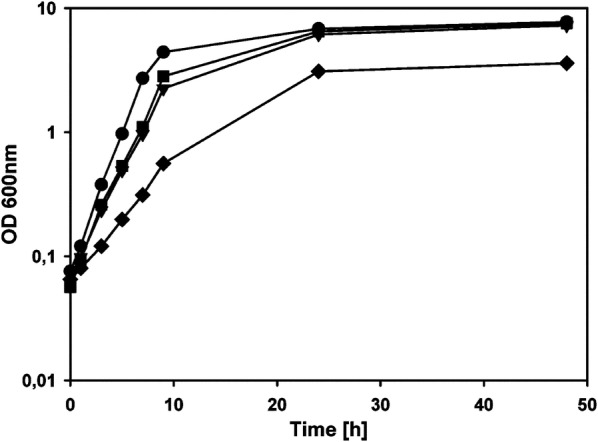
Growth of *E. coli* LJ110 in minimal media with glycerol and different l-PAPA concentrations. The cells were cultivated at 37 °C in the absence of l-PAPA (circles), or in the presence of 4 mM (squares), 8 mM (triangles), or 33 mM of l-PAPA (diamonds), respectively. Cultivations were performed twice and the mean values are given

As l-PAPA is a non-proteinogenic amino acid for wildtype *E. coli* cells, we wanted to know first whether it shows any inhibitory or toxic effects on growth. Therefore, cells of *E. coli* K-12 wildtype strain LJ110 (W3110) were grown in minimal media with glycerol as sole carbon source and synthetic l-PAPA was added at varying concentrations. As can be seen in Fig. 2, the growth rate in the presence of 4 or 8 mM l-PAPA was slightly reduced (µ = 0.43 h^−1^ and µ = 0.39 h^−1^, respectively) when compared to growth in the absence of l-PAPA (µ = 0.47 h^−1^); however, very similar final optical densities (OD_600_ values) were reached after 24 h of incubation. At a concentration of 33 mM l-PAPA (5.9 g l^−1^), however, the formation of biomass was impaired and the growth rate was conceivably reduced to µ = 0.24 h^−1^. Thus we reasoned that l-PAPA production in recombinant *E. coli* strains could be proceeded without serious toxicity problems caused by the novel product.

We decided to establish a synthetic pathway leading to l-PAPA by construction of a strain which carries the genes from different donor organisms (“metabolic grafting”). From previous work in our group [2, 41], the gene encoding 4-amino-4-deoxychorismate synthase from the PABA biosynthesis route of *C. glutamicum* (*pabAB*_*Cgl*_) was available in recombinant form and under the control of an IPTG-inducible P*tac* promoter on plasmid pC53 (see Table 1). We preferred the *pabAB*_*Cgl*_ gene over the two separate genes *pabA* and *pabB* which reside in the *E. coli* chromosome, as the plasmid-borne combination of the latter genes had shown less active enzyme [41]. To complete the intended l-PAPA pathway, genes *papB* and *papC* were added. *PapB* and *papC* were custom-synthesized based upon the gene sequences of *S. venezuelae* (*papB*, 4-amino-4-deoxy-chorismate mutase; *papC*, 4-amino-4-deoxyprephenate dehydrogenase) and codon optimized for expression in *E. coli* (GeneOptimizer, Thermo Fisher/GeneArt, Regensburg, Germany). The two genes were then cloned together as a cassette with *pabAB*_*Cgl*_ to yield plasmid pC53BC (see Table 1, Additional file 1: Figure S4).

**Table 1:** List of *E. coli* strains, plasmids and oligonucleotides

Strains	Characteristics	Reference/origin
*E. coli* DH5α	*φ80lacZΔM15 Δ(lacZYA*-*argF)U169 recA1 endA1 hsdR17(rK*−, *mK *+*) phoA supE44 λ*− *thi*-*1 gyrA96 relA1*	Invitrogen
*E. coli* LJ110	Wildtype W3110 (F−, λ−, IN (*rrnD*-*rrnE*) 1, *rph*-1)	[53]
*E. coli* FUS 4	LJ110 Δ(*pheA*-*tyrA*-*aroF*) Δ*lac*::P_*tac*_::*aroFBL*+	[15]
*E. coli* FUS4.7	FUS4 Δ*rbs*::P_*tac*_::*glpX*+ Δ*gal*::P_*tac*_::*tktA*+	[15]
*E. coli* FUS4.7R	FUS4.7Δ*tyrR::FRT*	This study
Plasmids		
pJNT522	P_*tac*_, RBS, Kan^R^	This study, see Additional file 1
pJNT-*aroFBL*	P_*tac*_::*aroF*_*E.c*._, *aroB*_*E.c*_, *aroL*_*E.c*._, Kan^R^	This study
pJF119EH	P_*tac*_, RBS, Amp^R^, *lacI*	[54]
pC53	pJF119EH, P_*tac*_::*pabAB*_*C.g*._, Amp^R^, *lacI*	[41]
pC53BC	pJF119EH, P_*tac*_::*pabAB*_*C.g*._-*papBC*_*S.v*._, Amp^R^, *lacI*	This study
pMK-*papBC*	Cloning vector including the codon optimized genes of *papBC*_*S.v*_, Kan^R^	Thermo Fisher (GeneArt) custom synthesis
pKD46	λ Red disruption system (γ, β, *exo* under control of P_*araBAD*_), Amp^R^	[55]
pCP20	FLP+, λ_cI857_+, λ ρR Rep^ts^, Amp^R^, Cm^R^	[56]
pCAS30-FRT-*cat*-FRT	pJF119ΔN, *P. ananatis crtE* gene, FRT-sites, Amp^R^, Cm^R^, source of *cat*-gene cassette	[57]
pJeM2	*rhaR rhaS rhaPBAD*, eGFP, mob Kan^R^	[58]

Primers	Sequence	
Del-*tyrR*-Fw	5′-ATCATCATATTAATTGTTCTTTTTTCAGGTGAAGGTTCCCTGTGTAGGCTGGAGCTGCTTCG-3′	
Del-*tyrR*-Rw	5′-AATATGCCTGATGGTGTTGCACCATCAGGCATATTCGCGCCATATGAATATCCTCCTTAG-3′	
Ko-*tyrR*-Fw	5′-TGACGGCACGACTCGGGATTAAAG-3′	
Ko-*tyrR*-Rw	5′-AACAACGTTATCACCCTC TCCACT-3′	

### l-PAPA production by recombinant *E. coli* strains

Expression of the gene cassette from pC53BC was first studied in the background of the wildtype strain *E. coli* LJ110. Cells were cultivated in shake flasks in minimal medium with glycerol (5 g l^−1^) and induction was by addition of IPTG (0.5 mM final concentration). This already led to l-PAPA accumulation of about 43 ± 2 mg l^−1^ in the supernatant whereas no l-PAPA was detected in the control strain (see Table 2). This proved that l-PAPA can be formed in conceivable amounts by *E. coli* cells and is in line with former observations with *E. coli* strains that were constructed in different manners [24, 43].

**Table 2:** Formation of l-PAPA in *E. coli* wild-type strain with different plasmid combinations

Plasmid combination	Final OD (600 nm)	Yield l-PAPA/glycerol (g g^−1^)	Final l-PAPA titer (mg l^−1^)
pJF119EH/pJNT522	5.89 ± 0.35	0	0
pC53BC/pJNT522	5.01 ± 0.95	0.01	43.2 ± 2
pC53BC/pJNT-*aroFBL*	4.75 ± 0.85	0.02	86.6 ± 4

*E. coli* LJ110 wild-type strain was transformed with two different and compatible IPTG-inducible plasmids (control plasmids pJF119EH and pJNT522) to study the effects of the presence of *pabAB*, *papBC*, and *aroFBL* genes, respectively. pC53BC carries *pabAB* and *papBC* as a gene cassette under the control of a P*tac* promoter. To study the effect of enhanced flux through the aromatic pathway, aroFBL were cloned onto pJNT522 plasmid. Biomass yields after 48 h of cultivation in minimal media with 5 g l^−1^ glycerol are shown as OD_600_ values. The cultivations were performed in triplicate and mean values and standard deviations are given

It is well-known that overexpression of the *aroF*, *aroB*, and *aroL* genes enhances formation of chorismate-derived compounds [3, 15, 59]. We therefore analyzed the effect of plasmid-borne overexpression of the genes *aroF* (DAHP synthase), *aroB* (dehydroquinate synthase) and *aroL* (shikimate kinase) present as a cassette on a second vector (pJNT522; Kan^R^, see Table 1 and Additional file 1: Figure S1) which is compatible with pC53BC. The additional IPTG-induced expression of *aroFBL* doubled the product titer after 48 h to 86.6 ± 3.7 mg l^−1^ (Table 2), most likely due to an improved provision of shikimate precursors for l-PAPA formation.

Next, to avoid an undesired loss of chorismate to l-Phe and l-Tyr formation (Fig. 1), a *pheA*-*tyrA*-*aroF* deletion mutant of LJ110 was used as host strain. This strain (FUS 4) is a double auxotroph for l-Phe and l-Tyr; it had been successfully used previously for the production of l-Phe; in addition, strain FUS4 carries a chromosomally inserted copy of the *aroFBL* cassette [15]. This second generation strain showed improved l-PAPA formation in shake flasks (minimal media with glycerol and growth supplementation by l-Phe and l-Tyr; see Table 3). The l-PAPA titer was raised to about 200 mg l^−1^ with a yield of ca. 0.04 g l-PAPA/g of glycerol compared to *E. coli* LJ110 (Table 2).

**Table 3:** Comparison of l-PAPA yields in next generation strains

*E. coli* strains	Final OD (600 nm)	Yield l-PAPA/glycerol (g g^−1^)	Final l-PAPA titer (mg l^−1^)
FUS 4/pC53BC/pJNT-*aroFBL*	3.77 ± 0.25	0.04	202.7 ± 4.5
FUS 4.7/pC53BC/pJNT-*aroFBL*	3.66 ± 0.13	0.09	449.4 ± 29.4
FUS 4.7R/pC53BC/pJNT-*aroFBL*	3.86 ± 0.14	0.11	534.1 ± 24.1

Double auxotroph strains carrying two plasmids (pC53BC and pJNT-*aroFBL*) were grown in shake flasks in minimal media with 5 g l^−1^ glycerol and supplemented by l-Phe and l-Tyr (0.04 g l^−1^ each) and appropriate antibiotics. Induction with IPTG (0.5 mM final concentration) was at OD_600_ of ca. 0.6 and strains were further incubated until a total cultivation time of 48 h. The cultivations were performed in triplicate and mean values and standard deviations are given

*Escherichia coli* FUS4.7, a FUS4 derivative which has additional chromosomal copies of *tktA* (transketolase A) and *glpX* (fructose 1,6-bisphosphate phosphatase), respectively, had turned up as an improved l-Phe producer before [15]. It was therefore used as recipient for pC53BC and pJNT-*aroFBL* to study its l-PAPA productivity. Indeed, the titer further increased to 449 ± 29 mg l^−1^ and the yield was more than double in comparison to FUS 4 with the same plasmid combination (see Table 3).

Finally, the gene for the transcriptional regulator, *tyrR* [60], was deleted. Such a deletion had been shown to confer a positive effect for aromatic compound production in engineered *E. coli* strains [61–66]. Deletion of *tyrR* in FUS 4.7 led to strain FUS4.7R. When transformed with plasmids pC53BC and pJNT-*aroFBL* this combination resulted in a slightly increased titer of l-PAPA (534 ± 24 mg l^−1^) and a yield of ca. 11% l-PAPA/glycerol (g g^−1^) (Table 3). The biomass yield of the three strains in shake flasks was about the same.

### Fed-batch shake flask cultivation with *E. coli* FUS4.7R/pC53BC/pJNT-*aroFBL*

**Fig. 3:**
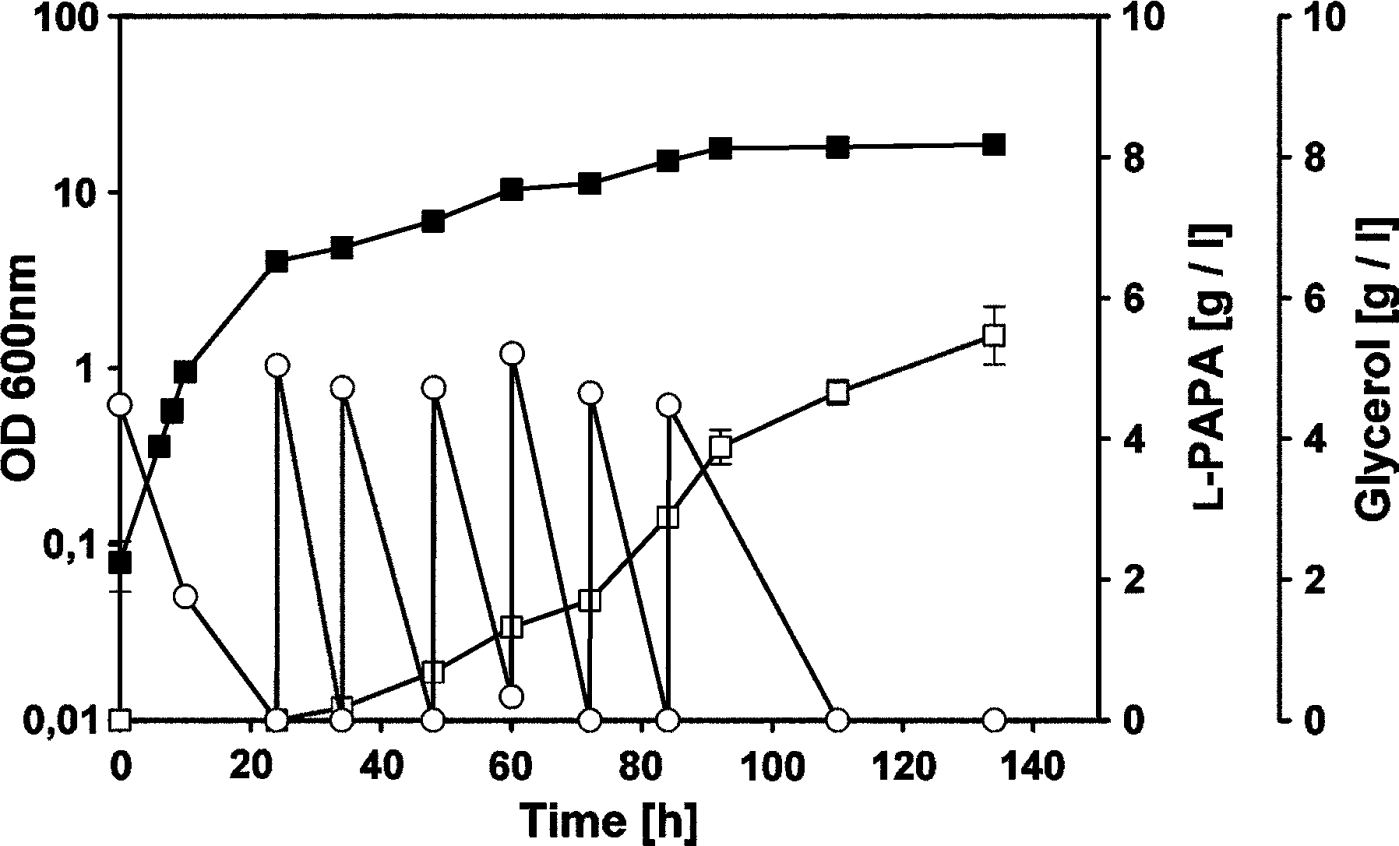
Glycerol fed-batch cultivation of *E. coli* FUS4.7 R/pC53BC/pJNT-*aroFBL* in shake flasks. The initial glycerol concentration was 5 g l^−1^. After 24 h of cultivation the cultures were induced with 0.5 mM IPTG (final concentration) and the shake flasks were transferred from 37 to 30 °C before adding pulses of ~ 5 g l^−1^ of glycerol every 12 h. To adjust the pH, sodium bicarbonate (30 mM) was added every 24 h as well as 15 mM of ammonium sulfate. The concentrations of glycerol (empty circles) and, l-PAPA (empty squares) were determined by HPLC. OD_600_ values are presented as filled squares. The cultivations were performed in triplicate and the mean values and standard deviations are given

These encouraging results from shake flask cultivations prompted us to study growth behavior and l-PAPA production in fed-batch condition. We chose the best producer, so far, *E. coli* FUS4.7R/pC53BC/pJNT-*aroFBL*. After pre-incubation and appropriate IPTG induction in shake flask with a starting concentration of 5 g l^−1^ of glycerol at 37 °C, temperature was lowered to 30 °C and glycerol (~ 5 g l^−1^) was added in intervals of 12 h. To roughly adjust for the pH, sodium bicarbonate was added (30 mM) every 24 h, as well as 15 mM ammonium sulfate as a nitrogen source to allow formation of l-PAPA. As a result, a total of 33.3 g l^−1^ glycerol was consumed in 134 h of cultivation (Fig. 3) and a titer of 5.47 ± 0.41 g l^−1^ of l-PAPA was detected in the supernatant; this corresponds to a yield of 16% l-PAPA/glycerol (g g^−1^).

### Fed-batch fermentation in a bioreactor

**Fig. 4:**
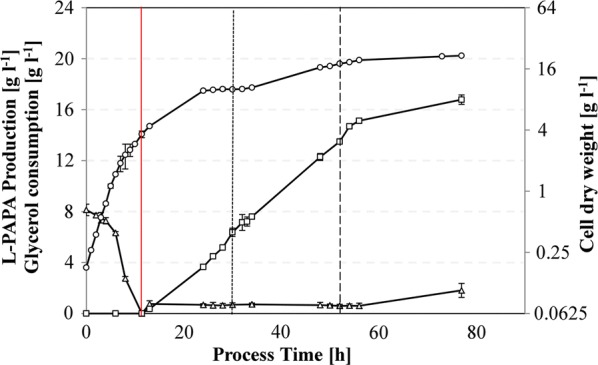
Fed-batch production of l-PAPA in a 30 l bioreactor. Cell dry weight (CDW, empty circle), glycerol consumption (g l^−1^, empty triangle) and l-PAPA concentration (g l^−1^, empty square) were monitored for *E. coli* FUS4.7R/pC53BC/pJNT-*aroFBL*. The strain was grown in minimal media with an initial concentration of 8.1 g l^−1^ glycerol. The glycerol feed started after 11.4 h (red solid line, first pulse of l-Phe and l-Tyr and induction with 0.5 mM IPTG) and the feed was adjusted to maintain a concentration of 0.6–1.0 g l^−1^ glycerol. A second pulse of l-Phe and l-Tyr (dotted line) and a third pulse of l-Phe and l-Tyr (dash line) were fed to allow biomass formation. The result of one fermentation is presented with mean values and deviations of three technical replicates

In order to scale up L-PAPA production under controlled conditions, we cultivated *E. coli* FUS4.7R/pC53BC/pJNT-*aroFBL* cells in an aerated, pH-controlled 30 l stirred-tank reactor with minimal medium and glycerol as sole carbon source. The starting volume for the batch was 8 l and the final volume (after fed-batch) of 12.25 l. Details of the fermentation procedure are given in the Materials and Methods section. The feeding started after the initially added glycerol (8.1 g l^−1^) was consumed. Glycerol was then fed to maintain a concentration between 0.6 and 1.0 g l^−1^. Additionally, l-Phe and l-Tyr were added in two pulses to allow biomass formation. After 77 h, a final cell dry weight of 21.6 g l^−1^ was reached and 131.7 g l^−1^ of glycerol had been consumed (see Fig. 4). A total concentration of 16.78 g l^−1^ l-PAPA was produced by then. This equals a yield of 0.13 l-PAPA/glycerol (g g^−1^) and a space–time-yield of l-PAPA formation over the whole process of 0.22 g l^−1^ h^−1^. The total amount of l-PAPA produced during the 77 h of fermentation was 205 g. The produced l-PAPA was analyzed by MS and the obtained mass was in excellent agreement with the commercially available l-PAPA (Additional file 2).

To our knowledge, this is the highest l-PAPA titer which has been reached with *E. coli* cells.

## Discussion

Expansion of the chorismate pathway of *E. coli* to produce a variety of non-standard aromatic compounds has already been very successful in many cases. Pioneering work has been done by the group of Frost and others already in the 1990s [61–64, 66]. Thereafter, processes have been published for *p*-hydroxybenzoic acid [61, 65], *p*-aminobenzoic acid [40, 42], protocatechuic acid and catechol [65], vanillin [67, 68], anthranilic acid [69], δ-tocotrienol (a vitamin E compound, [70]), phenol [71–74], salicylic acid and muconic acid [5, 75], styrene, cinnamic acid and hydroxylated derivatives thereof [7, 76–80], tyrosol [81], (hydroxyl)phenyllactic acid, tyramine and other l-Phe- or l-Tyr-derived compounds [76], rosmarinic acid and flavonoids [82, 83], antitumor drugs like violacein and deoxyviolacein [84], several plant alkaloids [85], or even opiates like thebaine, reticuline or hydrocodone [86]. Further examples may be found in recent review articles [79, 87, 88].

These approaches most often have in common that heterologous genes from other donor organisms and from other biosynthetic pathways are used for transplantation into the genome of recipient strains such as *E. coli*. These recipients often have been engineered for a fortified provision of chorismate from the general aromatic amino acid pathway [61, 66, 69, 89]. We propose to classify this subdivision of “metabolic engineering” [90] as “metabolic grafting” [3] as it may be metaphorically likened to the well-known and centuries old procedure in plant husbandry which is termed “grafting”. Therein, for example, a fledgling twig from a precious fruit tree is merged (pruned) with a robust stem of another tree (which may be of another plant species), resulting in strong fruit bearing by the grafted-upon plant (see for example the case of using North American vine as rootstock for many rare and precious European wines to overcome the *Phylloxera* vine pest in the late nineteenth century [91]). In analogy, the transfer of such a grafting procedure to the expansion of the chorismate pathway in microorganisms thus implies cloning and expression of heterologous genes to allow production of non-standard products. Whereas the proteinogenic aromatic amino acids l-Phe, l-Tyr, and l-Trp can be produced by recombinant *E. coli* strains even without the introduction of foreign genes ([3, 89, 92], non-standard and non-proteinogenic aromatic amino acids require such a grafting procedure. Examples are the production of l-homophenylalanine by introduction of genes from the cyanobacterium *Nostoc punctiforme* for the chain elongation of l-Phe [93], improved production of l-3,4-dihydroxyphenylalanine (l-DOPA) from *E. coli* grown on glucose [94, 95], or the production of d-phenylglycine via phenylpyruvic acid and mandelic acid [96].

Following the initial work of the Schultz group on l-PAPA formation in recombinant *E. coli* strains using the genes *papABC* from *S. venezuelae* [43], Takaya and coworkers recently presented a genetically modified *E. coli* to produce l-PAPA from glucose by introduction of *papABC* genes from *P. fluorescens* [24]. A high titer of 4.4 l-PAPA g l^−1^ with a production yield of 17% (g g^−1^) from glucose was achieved in minimal media supplemented with tryptone (5 g l^−1^) and yeast extract (2.5 g l^−1^) in a bioreactor [24].

The aim of the present study was to produce the high value compound l-PAPA with genetically engineered *E. coli* strains from glycerol, a renewable carbon source (for a recent review see [48]). Glycerol has become a favorable carbon source as it is a byproduct of biodiesel production [15, 48, 52, 97]. The advantage to use glycerol instead of glucose is that there is no need of stoichiometric amounts of phosphoenolpyruvate (PEP) for its uptake into *E. coli* cells, whereas the PTS- dependent glucose uptake needs PEP [98]. PEP and E4P are important precursors of the initial reaction of shikimate pathway to form DAHP by the DAHP synthase [99]. Furthermore, glycerol (a C3 unit), compared to glucose (a C6 unit), has a higher reduction potential per C3 unit which in turn could yield higher biomass and product formation [15, 48, 100–102]. The precursor supply is a pivotal parameter to improve production of compound with origin in the shikimate pathway [17]. Our group had shown in previous studies [15, 18] that the improved supply of E4P by integration of additional gene copies of the fructose 1,6-bisphosphate phosphatase (*glpX*) and transketolase (*tktA*) increased the yield for l-Phe production [15, 18]. Therefore we applied this knowledge here and found that l-PAPA synthesis also profited from these genetic alterations. The extra gene copies in FUS 4.7 led to a doubled product titer of about 0.45 ± 0.03 g l^−1^ in batch culture shake flasks after 48 h compared to the parent strain FUS4 (0.20 ± 0.01 g l^−1^) in minimal medium with 5 g l^−1^ glycerol (Table 2).

Moreover, we analyzed the effect of a *tyrR* deletion in the recombinant *E. coli* FUS4.7. The transcriptional regulon of TyrR is well characterized in *E. coli* [60]. TyrR downregulates as transcriptional repressor the genes *aroF*, *aroG*, *aroL*, *tyrP*, *aroP*, *tyrA* and *tyrB* in the presence of l-Tyr [60, 103]. We observed a beneficial effect on l-PAPA production by a *tyrR* deletion in *E. coli* FUS4.7R compared to *E. coli* FUS4.7. The yield was increased by ~ 18% from 0.09 l-PAPA/glycerol (g g^−1^) to 0.11 l-PAPA/glycerol (g g^−1^) in batch cultivation. This result is in good agreement with previous studies [76, 92, 94]. It is likely that the positive effect of a *tyrR* deletion relies on the increased transcriptional expression and resulting increased specific activities for members of the TyrR regulon.

By changing the cultivation condition of *E. coli* FUS4.7R to a fed-batch cultivation in shake flasks, we improved the total yield to 0.16 l-PAPA/glycerol (g g^−1^) and reached a titer of about 5.47 ± 0.41 g l^−1^ l-PAPA after 134 h. This titer is already higher than the 4.4 g l^−1^ obtained found by the Takaya group in minimal media with glucose as main C source (plus added tryptone and yeast extract) [24]. However, as the cultivation in fed-batch shake flasks is not optimal in term of oxygen supply and pH stability it may be advantageous to perform the cultivation in a pH- and pO_2_-controlled bioreactor. Indeed, we found that during a run of 77 h, 131.7 g l^−1^ of glycerol were consumed and a total of 205 g of l-PAPA were produced (16.78 g l^−1^). Only small amounts of acetate (< 2 g l^−1^) and no lactate as by-products were detected during fermentation. This corresponds to a yield of 0.13 g l-PAPA per g of glycerol, slightly lower than in the fed-batch shake flask cultures. The reached titer in the bioreactor showed that l-PAPA production at higher concentration is still feasible although the growth of *E. coli* LJ110 was impaired at 5.9 g l^−1^ (Fig. 2). In comparison with other products of “extended shikimate pathway” [7], the presented data on l-PAPA are in the same range as *p*-aminobenzoate from glucose in recombinant *E. coli* (4.8 g l^−1^ in 48 h; yield of 0.16 g g^−1^ [79]) but below anthranilate (*o*-aminobenzoate: 14 g l^−1^ in 34 h on glucose with a yield of 0.2 g g^−1^ [69]) where both products, however, need only one ammonium equivalent for their production.

To further improve the E4P precursor supply it can be considered to further increase the number of gene copies in the *E. coli* genome as only one additional copy of *tktA* and *glpX* was inserted. The enhanced flux through the shikimate pathway by overexpression of plasmid-borne *aroFBL* genes showed a beneficial effect in our study. Other studies have shown that the overexpression of the other shikimate pathway genes (*aroA*, *C*, *D* and *E*) as well improved the formation of l-Tyr which is derived from chorismate [14]. It is likely that a positive effect can be also observed for the l-PAPA production. This improved route to l-PAPA based upon a simple medium with a sustainable carbon source which is currently in ample supply from biodiesel production and which does not compete with human nutrition, opens up the possibility to produce an interesting building block in larger scale.

## Conclusion

This study demonstrated that *E. coli* is a good chassis strain for l-PAPA production using glycerol as an alternative sole carbon source. We constructed by metabolic grafting a de novo pathway for l-PAPA in *E. coli*. By improving the E4P precursor supply and the increased flux through the shikimate pathway both l-PAPA titer and yield were augmented.

## Methods

Chemicals, antibiotics, buffer components, culture media and analytical standards, used in this study were purchased from AppliChem GmbH (Darmstadt, Germany), Carl Roth GmbH (Karlsruhe, Germany), or Sigma-Aldrich/Fluka (Taufkirchen, Germany) and were of the highest available purity. l-PAPA was purchased from Sigma-Aldrich Chemie GmbH (Taufkirchen, Germany).

### Bacterial strains, plasmids and cultivation conditions

All bacterial strains, plasmids, and oligonucleotides used in this study are listed in Table 1. For the cultivation of *E. coli*, lysogeny broth (LB) was used as complex media [104]. The minimal media (MM) contained 3 g l^−1^ KH_2_PO_4_, 12 g l^−1^ K_2_HPO_4_, 5 g l^−1^ (NH_4_)_2_SO_4_, 0,3 g l^−1^ MgSO_4_·7H_2_O, 0,1 g l^−1^ NaCl, 0,1125 g l^−1^ FeSO_4_·7H_2_O/Na citrate 15 ml (from the solution of 7.5 g l^−1^ FeSO_4_ and 100 g l^−1^ sodium citrate), 0,015 g l^−1^ CaCl_2_·2H_2_O, 7.5 µg l^−1^ thiamine HCl, 0,04 mg ml^−1^ l-Phe, 0,04 mg ml^−1^ l-Tyr and 5 g l^−1^ glycerol as sole carbon source [15]. Ampicillin sodium salt (100 mg l^−1^) and/or kanamycin sulfate (50 mg l^−1^) was added when appropriate.

*Escherichia coli* strains were grown in 250 ml shake flasks filled with 20 ml of medium at 37 °C and with 150-rpm agitation. Overnight cultures were used as inocula (1% v/v). Induction was with 0.5 mM isopropyl-β-d-thiogalactopyranoside (IPTG, final concentration) at an OD at 600 nm (OD_600_) of about 0.6. Samples were taken and centrifuged at 22,000*g* for 10 min. The supernatants were removed and stored at − 20 °C until further use. The glycerol fed-batch cultivation was performed in 250 ml shake flasks with 20 ml media. The initial glycerol concentration was 5 g l^−1^. Cultures were inoculated with an overnight grown culture. After 24 h of cultivation, 0.5 mM IPTG (final concentration) was added to the cells and the shake flasks were transferred from 37 to 30 °C before starting to add ~ 5 g l^−1^ glycerol to the culture every 12 h until 84 h. Furthermore, every 24 h, 30 mM sodium bicarbonate was added to adjust the pH and 15 mM ammonium sulfate was added as ammonium source. Samples were taken and centrifuged at 22,000*g* for 10 min. The supernatants were removed and stored at − 20 °C until use.

### Fed-batch fermentation in a 30 l bioreactor

A 30 l stirred-tank reactor (Bioengineering, Wald, Switzerland) with a batch volume of 8 l and a final volume of 12.25 l was used and a minimal medium with glycerol as sole carbon source. The concentration of supplementation compounds was increased 1.5-fold, e.g. supplementation was with 60 mg l^−1^ l-Phe, 60 mg l^−1^ of l-Tyr. The concentration of antibiotics was kept at 100 mg l^−1^ ampicillin sodium salt and 50 mg l^−1^ kanamycin sulfate. The working conditions were pH 7.0 (controlled by the addition of 15% ammonia solution), 30% oxygen saturation with a reactor pressure of 500 hPa above atmospheric pressure (starting with pO_2_ ~ 100% at 350 rpm with an aeration rate of 4 l min^−1^; when reaching 30% oxygen saturation, the stirring rate was adjusted). A preculture (1.2 l grown in shake flasks with the same medium) which was in the exponential growth in shake flasks, was used to inoculate the fermentation (starting CDW ~ 0.18 g l^−1^) t 37 °C. 11.75 h after inoculation IPTG (0.5 mM), l-Tyr (0.36 mM) and l-Phe (0.33 mM) were added and the temperature was shifted from 37 to 30 °C. The initial glycerol concentration was about 8.1 g l^−1^. The glycerol feed was started after the initial glycerol was depleted (indicated by a rise in pO_2_). Glycerol was fed to maintain a concentration between 0.6 and 1.0 g l^−1^ during cultivation. If needed, antifoam agent (Struktol J647) from Schill Seilacher (Hamburg) was added to the culture. Additionally, l-Phe and l-Tyr were added in two pulses (34 h after inoculation 0.5 mM l-Phe and l-Tyr; 56 h after inoculation 0.3 mM l-Phe and l-Tyr) to allow biomass formation.

### DNA manipulations

For plasmid constructions, *E. coli* strain DH5α was used. Standard molecular biology methods were applied [104]. DNA containing the genes *papBC* from *S. venezuelae* was custom synthesized (Thermo Fisher/GeneArt, Regensburg, Germany) and codon optimized for *E. coli* to give pMK-*papBC* (see Table 1); this plasmid DNA was then restricted with *Bgl*II and *Bam*HI. A resulting fragment containing *papBC* was isolated and ligated with a *Bam*HI restricted pC53 vector (based on pJF119 EH vector [44] which already carries the *pabAB* genes from *C. glutamicum* [41]) to obtain pC53BC (see Additional file 1: Figure S4). The recombinant genes are under the control of the *lacI*^q^/P_*tac*_ promoter. The sequence and direction of *papBC* was verified by sequencing (GATC Biotech AG, Konstanz, Germany). Plasmid pJNT-*aroFBL* is based on vector pJNT522 which in turn is derived from pJeM2 [58] and pJF119EH. Construction of pJNT522 vector is described in the Additional file 1. In brief, pJNT522 contains a fragment of pJeM2 plasmid carrying the *mob* region, origin of replication, and kanamycin resistance gene as well as the P*ta*c promoter, MCS and *rrnB* T1T2 terminator of transcription from the pJF119EH vector. pJNT522 is compatible with pJF119EH- derived plasmids. The subcloning of *aroFB* and *aroL* genes of *E. coli* and ligation on vector pJNT522 to yield pJNT-*aroFBL* is described in the Additional file 1.

### Deletion of *tyrR* gene from the *E. coli* chromosome

The deletion of the gene *tyrR*, encoding the regulator of the *tyr* regulon, was carried out according to a λred-recombineering method [55]. A linear DNA fragment containing the FRT-flanked chloramphenicol resistance (*cat*) cassette was amplified from plasmid pCAS30-FRT-*cat*-FRT [57] using the primer pair Del*tyrR*Fw/-Rw (see Table 1 for sequences). The thus obtained amplified linear DNA was introduced by electroporation into electrocompetent *E. coli* FUS4.7 cells that carried the Red recombinase expression vector pKD46 [55]. After confirmation of the FRT-*cat*-FRT integration by colony PCR with the primer pair Ko-tyrRFw/-Rw (see Table 1), the *cat* marker was removed by transient expression of a FLP recombinase from plasmid pCP20 [56] to eventually generate the *tyrR* deletion strain, FUS4.7R. Verification of the disruption was performed using colony PCR with primers up- and downstream of disrupted regions (Ko-*tyrR*-Fw/-Rw). Finally, cells were grown at 42 °C to remove the temperature-sensitive replicons, pCP20 or pKD46, respectively [55].

### Analytical methods

Growth of *E. coli* cells was followed by measuring OD_600_ in a UV–vis spectrophotometer (Cary 50 Bio, Agilent Technologies). The formation of l-PAPA was routinely analyzed and quantified by high-performance liquid chromatography (HPLC, 1260 Infinity series, Agilent Technologies) with a diode array detector (1260 Infinity series, Agilent Technologies) at a wavelength of 210 nm. A Prontosil C18 column (250 × 4 mm, CS-Chromatographie Services GmbH, Langerwehe, Germany) was used for separation at 40 °C. The mobile phase containing 40 mM Na_2_SO_4_ (adjusted at pH ~ 2.7 with methane sulfonic acid) was used with a flow rate of 1 ml min^−1^. Glycerol concentrations were determined by HPLC with a refractive index detector (1260 Infinity series, Agilent Technologies) and an Organic Acid column (300 × 8 mm, CS-Chromatographie Services GmbH, Langerwehe Germany). An isocratic flow of 0.6 ml min ^−1^ of 5 mM H_2_SO_4_ was applied at 40 °C. The produced L-PAPA was analyzed by mass spectrometry. It was determined by using an Agilent 6130 mass spectrometer system with electron spray ionization (Agilent Technologies; Germany).

## Additional files

**Additional file 1.** Construction and sequences of vectors

**Additional file 2.** Comparison of mass spectra of commercially available L-PAPA (A) with fermentatively produced L-PAPA from *E. coli* FUS4.7R/pC53BC/pJNT*aroFBL* (B). The mass spectra were recorded with an Agilent 6130 mass spectrometer system with electron spray ionization in positive mode (Agilent Technologies; Germany). In both measurements the L-PAPA+H^+^ mass of 181 was detected.

## Abbreviations

ADC: 4-amino-4-deoxychorismate
ADP: adenosine diphosphate
α-KG: alpha-ketoglutarate
Amp: ampicillinAspC aspartate aminotransferase
AroB: dehydroquinate synthase
AroF: DAHP synthase
AroL: shikimate kinase
ATP: adenosine triphosphate
DAHP: 3-deoxy-d-arabino-heptulosonate 7-phosphate
DHAP: dihydroxyacetone phosphate
DHQ: dehydroquinate
E4P: erythrose-4-phosphate
F6P: fructose-6-phosphate
FBP: fructose-1,6-bisphosphate
G3P: glycerol-3-phosphate
GAP: glyceraldehyde-3-phosphate
GlpD: glycerol-3-phosphate dehydrogenase
GlpF: glycerol facilitator
GlpK: glycerol kinase
Glu: l-glutamate
Gln: l-glutamine
GlpX: fructose-1,6-bisphosphatase
IPTG: isopropyl-β-d-thiogalactopyranoside
Kan: kanamycin
l-PAPA: *para*-amino-l-phenylalanine
l-Phe: l-phenylalanine
l-Trp: l-typtophan
l-Tyr: l-tyrosine
MQ: menaquinone
OD: optical density
PABA: para-aminobenzoate
PabAB: 4-amino-4-deoxychorismate synthase
PapB: 4-amino-4-deoxychorismate mutase
PapC: 4-amino-4-deoxyprephenate dehydrogenase
PAPP: *para*-aminophenylpyruvate
PEP: phosphoenolpyruvate
PTS: PEP-dependent sugar:phosphotransferase system
TCA: tricarboxylic acid cycle
TktA: transketolase A
TyrB: aromatic aminotransferase
UQ: ubiquinone

## References

[1] Gibson F, Jackman LM (1963). Structure of chorismic acid, a new intermediate in aromatic biosynthesis. Nature.

[2] Bongaerts J, Esser S, Lorbach V, Al-Momani L, Muller MA, Franke D, Grondal C, Kurutsch A, Bujnicki R, Takors R (2011). Diversity-oriented production of metabolites derived from chorismate and their use in organic synthesis. Angew Chem Int Ed Engl.

[3] Sprenger GA (2007). From scratch to value: engineering *Escherichia coli* wild type cells to the production of l-phenylalanine and other fine chemicals derived from chorismate. Appl Microbiol Biotechnol.

[4] Berry A (1996). Improving production of aromatic compounds in *Escherichia coli* by metabolic engineering. Trends Biotechnol.

[5] Noda S, Shirai T, Oyama S, Kondo A (2016). Metabolic design of a platform *Escherichia coli* strain producing various chorismate derivatives. Metab Eng.

[6] van Beilen JWA, Hellingwerf KJ (2016). All three endogenous quinone species of *Escherichia coli* are involved in controlling the activity of the aerobic/anaerobic response regulator ArcA. Front Microbiol.

[7] Lee JH, Wendisch VF (2017). Biotechnological production of aromatic compounds of the extended shikimate pathway from renewable biomass. J Biotechnol.

[8] Young IG, Langman L, Luke RK, Gibson F (1971). Biosynthesis of the iron-transport compound enterochelin: mutants of *Escherichia coli* unable to synthesize 2,3-dihydroxybenzoate. J Bacteriol.

[9] Mene-Saffrane L, DellaPenna D (2010). Biosynthesis, regulation and functions of tocochromanols in plants. Plant Physiol Biochem.

[10] Mast Y, Weber T, Golz M, Ort-Winklbauer R, Gondran A, Wohlleben W, Schinko E (2011). Characterization of the ‘pristinamycin supercluster’ of *Streptomyces pristinaespiralis*. Microb Biotechnol.

[11] Lin DR, Xiao MS, Zhao JJ, Li ZH, Xing BS, Li XD, Kong MZ, Li LY, Zhang Q, Liu YW (2016). An overview of plant phenolic compounds and their importance in human nutrition and management of type 2 diabetes. Molecules.

[12] Fernandez-Martinez LT, Borsetto C, Gomez-Escribano JP, Bibb MJ, Al-Bassam MM, Chandra G, Bibb MJ (2014). New insights into chloramphenicol biosynthesis in *Streptomyces venezuelae* ATCC 10712. Antimicrob Agents Chemother.

[13] Schuhmacher T, Loffler M, Hurler T, Takors R (2014). Phosphate limited fed-batch processes: impact on carbon usage and energy metabolism in *Escherichia coli*. J Biotechnol.

[14] Juminaga D, Baidoo EE, Redding-Johanson AM, Batth TS, Burd H, Mukhopadhyay A, Petzold CJ, Keasling JD (2012). Modular engineering of l-tyrosine production in *Escherichia coli*. Appl Environ Microbiol.

[15] Gottlieb K, Albermann C, Sprenger GA (2014). Improvement of l-phenylalanine production from glycerol by recombinant *Escherichia coli* strains: the role of extra copies of *glpK*, *glpX*, and *tktA* genes. Microb Cell Fact.

[16] Ikeda M (2006). Towards bacterial strains overproducing l-tryptophan and other aromatics by metabolic engineering. Appl Microbiol Biotechnol.

[17] Rodriguez A, Martinez JA, Flores N, Escalante A, Gosset G, Bolivar F (2014). Engineering *Escherichia coli* to overproduce aromatic amino acids and derived compounds. Microb Cell Fact.

[18] Weiner M, Trondle J, Albermann C, Sprenger GA, Weuster-Botz D (2017). Metabolic control analysis of l-phenylalanine production from glycerol with engineered *E. coli* using data from short-term steady-state perturbation experiments. Biochem Eng J.

[19] Min K, Park K, Park DH, Yoo YJ (2015). Overview on the biotechnological production of l-DOPA. Appl Microbiol Biotechnol.

[20] Cluis CP, Ekins A, Narcross L, Jiang H, Gold ND, Burja AM, Martin VJ (2011). Identification of bottlenecks in *Escherichia coli* engineered for the production of CoQ(10). Metab Eng.

[21] Xu W, Yang S, Zhao J, Su T, Zhao L, Liu J (2014). Improving coenzyme Q8 production in *Escherichia coli* employing multiple strategies. J Ind Microbiol Biotechnol.

[22] Kumar A, Tateyama S, Yasaki K, Ali MA, Takaya N, Singh R, Kaneko T (2016). (1)H NMR and FT-IR dataset based structural investigation of poly(amic acid)s and polyimides from 4,4′-diaminostilbene. Data Brief.

[23] Yamaguchi M, Saito R, Utsumi K, Ochiai A, Kawashima N, Tokuoka Y, Katoh A (2007). The nicotinic acid-p-aminophenylalanine-hydroxybenzoic acid triads induce apoptosis in human leukemia U937 cells. Heterocycles.

[24] Masuo S, Zhou S, Kaneko T, Takaya N (2016). Bacterial fermentation platform for producing artificial aromatic amines. Sci Rep.

[25] Suvannasara P, Tateyama S, Miyasato A, Matsumura K, Shimoda T, Ito T, Yamagata Y, Fujita T, Takaye N, Kaneko T (2014). Biobased polyimides from 4-aminocinnamic acid photodimer. Macromolecules.

[26] Bergel F, Stock JA (1954). Cyto active amino acid and peptide derivatives. I. Substituted phenylalanines. J Chem Soc.

[27] Jobdevairakkam C, Velladurai H. Process of making optically pure melphalan. WO2009117164A1. 2013.

[28] Dardenne GA, Larsen PO, Wieczorkowska E (1975). Biosynthesis of p-aminophenylalanine: part of a general scheme for the biosynthesis of chorismic acid derivatives. Biochim Biophys Acta.

[29] Chang Z, Sun Y, He J, Vining LC (2001). p-Aminobenzoic acid and chloramphenicol biosynthesis in *Streptomyces venezuelae*: gene sets for a key enzyme, 4-amino-4-deoxychorismate synthase. Microbiology.

[30] Blanc V, Gil P, Bamas-Jacques N, Lorenzon S, Zagorec M, Schleuniger J, Bisch D, Blanche F, Debussche L, Crouzet J, Thibaut D (1997). Identification and analysis of genes from *Streptomyces pristinaespiralis* encoding enzymes involved in the biosynthesis of the 4-dimethylamino-l-phenylalanine precursor of pristinamycin I. Mol Microbiol.

[31] Mast YJ, Wohlleben W, Schinko E (2011). Identification and functional characterization of phenylglycine biosynthetic genes involved in pristinamycin biosynthesis in *Streptomyces pristinaespiralis*. J Biotechnol.

[32] Schaffer JE, Reck MR, Prasad NK, Wencewicz TA (2017). beta-Lactone formation during product release from a nonribosomal peptide synthetase. Nat Chem Biol.

[33] Nollmann FI, Dauth C, Mulley G, Kegler C, Kaiser M, Waterfield NR, Bode HB (2015). Insect-specific production of new GameXPeptides in *Photorhabdus luminescens* TTO1, widespread natural products in entomopathogenic bacteria. ChemBioChem.

[34] Martinez-Farina CF, Robertson AW, Yin HM, Monro S, McFarland SA, Syvitski RT, Jakeman DL (2015). Isolation and synthetic diversification of jadomycin 4-amino-l-phenylalanine. J Nat Prod.

[35] Kawasaki Y, Aniruddha N, Minakawa H, Masuo S, Kaneko T, Takaya N (2018). Novel polycondensed biopolyamide generated from biomass-derived 4-aminohydrocinnamic acid. Appl Microbiol Biotechnol.

[36] Tateyama S, Masuo S, Suvannasara P, Oka Y, Miyazato A, Yasaki K, Teerawatananond T, Muangsin N, Zhou SM, Kawasaki Y (2016). Ultrastrong, transparent polytruxillamides derived from microbial photodimers. Macromolecules.

[37] Viswanathan VK, Green JM, Nichols BP (1995). Kinetic characterization of 4-amino 4-deoxychorismate synthase from *Escherichia coli*. J Bacteriol.

[38] Nichols BP, Seibold AM, Doktor SZ (1989). para-aminobenzoate synthesis from chorismate occurs in two steps. J Biol Chem.

[39] Stolz M, Peters-Wendisch P, Etterich H, Gerharz T, Faurie R, Sahm H, Fersterra H, Eggeling L (2007). Reduced folate supply as a key to enhanced l-serine production by *Corynebacterium glutamicum*. Appl Environ Microbiol.

[40] Kubota T, Watanabe A, Suda M, Kogure T, Hiraga K, Inui M (2016). Production of para-aminobenzoate by genetically engineered *Corynebacterium glutamicum* and non-biological formation of an N-glucosyl byproduct. Metab Eng.

[41] Kozak S. Mikrobielle Biosynthese von (3S,4R)-4-amino-3-hydroxycyclohexa-1,5-diencarbonsäure mit rekombinanten *Escherichia coli* Zellen: Molekulargenetische und biochemische Untersuchungen. Ph.D. Thesis, University of Stuttgart, Germany; 2006.

[42] Koma D, Yamanaka H, Moriyoshi K, Sakai K, Masuda T, Sato Y, Toida K, Ohmoto T (2014). Production of *p*-aminobenzoic acid by metabolically engineered *Escherichia coli*. Biosci Biotechnol Biochem.

[43] Mehl RA, Anderson JC, Santoro SW, Wang L, Martin AB, King DS, Horn DM, Schultz PG (2003). Generation of a bacterium with a 21 amino acid genetic code. J Am Chem Soc.

[44] He J, Magarvey N, Piraee M, Vining LC (2001). The gene cluster for chloramphenicol biosynthesis in *Streptomyces venezuelae* ISP5230 includes novel shikimate pathway homologues and a monomodular non-ribosomal peptide synthetase gene. Microbiology.

[45] Yanai K, Sumida N, Okakura K, Moriya T, Watanabe M, Murakami T (2004). Para-position derivatives of fungal anthelmintic cyclodepsipeptides engineered with *Streptomyces venezuelae* antibiotic biosynthetic genes. Nat Biotechnol.

[46] Hayashi H, Inoue K, Nagata T, Kuramitsu S, Kagamiyama H (1993). *Escherichia coli* aromatic amino acid aminotransferase: characterization and comparison with aspartate aminotransferase. Biochemistry.

[47] Onuffer JJ, Ton BT, Klement I, Kirsch JF (1995). The use of natural and unnatural amino acid substrates to define the substrate specificity differences of *Escherichia coli* aspartate and tyrosine aminotransferases. Protein Sci.

[48] Sprenger GA, Gosset G (2017). Glycerol as carbon source for production of added-value compounds. Engineering of microorganisms for the production of chemicals and biofuels from renewable resources.

[49] Richey DP, Lin EC (1972). Importance of facilitated diffusion for effective utilization of glycerol by *Escherichia coli*. J Bacteriol.

[50] Zwaig N, Kistler WS, Lin EC (1970). Glycerol kinase, the pacemaker for the dissimilation of glycerol in *Escherichia coli*. J Bacteriol.

[51] Weiner JH, Heppel LA (1972). Purification of the membrane-bound and pyridine nucleotide-independent l-glycerol 3-phosphate dehydrogenase from *Escherichia coli*. Biochem Biophys Res Commun.

[52] Almeida JR, Favaro LC, Quirino BF (2012). Biodiesel biorefinery: opportunities and challenges for microbial production of fuels and chemicals from glycerol waste. Biotechnol Biofuels.

[53] Bachmann BJ (1972). Pedigrees of some mutant strains of *Escherichia coli* K-12. Bacteriol Rev.

[54] Furste JP, Pansegrau W, Frank R, Blocker H, Scholz P, Bagdasarian M, Lanka E (1986). Molecular cloning of the plasmid RP4 primase region in a multi-host-range tacP expression vector. Gene.

[55] Datsenko KA, Wanner BL (2000). One-step inactivation of chromosomal genes in *Escherichia coli* K-12 using PCR products. Proc Natl Acad Sci USA.

[56] Cherepanov PP, Wackernagel W (1995). Gene disruption in *Escherichia coli*: TcR and KmR cassettes with the option of Flp-catalyzed excision of the antibiotic-resistance determinant. Gene.

[57] Vallon T, Ghanegaonkar S, Vielhauer O, Muller A, Albermann C, Sprenger G, Reuss M, Lemuth K (2008). Quantitative analysis of isoprenoid diphosphate intermediates in recombinant and wild-type *Escherichia coli* strains. Appl Microbiol Biotechnol.

[58] Jeske M, Altenbuchner J (2010). The *Escherichia coli* rhamnose promoter *rhaP*(*BAD*) is in *Pseudomonas putida* KT2440 independent of Crp-cAMP activation. Appl Microbiol Biotechnol.

[59] Gosset G (2009). Production of aromatic compounds in bacteria. Curr Opin Biotechnol.

[60] Pittard J, Camakaris H, Yang J (2005). The TyrR regulon. Mol Microbiol.

[61] Barker JL, Frost JW (2001). Microbial synthesis of *p*-hydroxybenzoic acid from glucose. Biotechnol Bioeng.

[62] Bongaerts J, Kramer M, Muller U, Raeven L, Wubbolts M (2001). Metabolic engineering for microbial production of aromatic amino acids and derived compounds. Metab Eng.

[63] Draths KM, Frost JW (1994). Environmentally compatible synthesis of adipic acid from d-glucose. J Am Chem Soc.

[64] Frost JW, Draths KM (1995). Biocatalytic syntheses of aromatics from d-glucose: renewable microbial sources of aromatic compounds. Annu Rev Microbiol.

[65] Pugh S, McKenna R, Osman M, Thompson B, Nielsen DR (2014). Rational engineering of a novel pathway for producing the aromatic compounds p-hydroxybenzoate, protocatechuate, and catechol in *Escherichia coli*. Process Biochem.

[66] Snell KD, Draths KM, Frost JW (1996). Synthetic modification of the *Escherichia coli* chromosome: enhancing the biocatalytic conversion of glucose into aromatic chemicals. J Am Chem Soc.

[67] Li K, Frost JW (1998). Synthesis of vanillin from glucose. J Am Chem Soc.

[68] Kunjapur AM, Tarasova Y, Prather KL (2014). Synthesis and accumulation of aromatic aldehydes in an engineered strain of *Escherichia coli*. J Am Chem Soc.

[69] Balderas-Hernandez VE, Sabido-Ramos A, Silva P, Cabrera-Valladares N, Hernandez-Chavez G, Baez-Viveros JL, Martinez A, Bolivar F, Gosset G (2009). Metabolic engineering for improving anthranilate synthesis from glucose in *Escherichia coli*. Microb Cell Fact.

[70] Albermann C, Ghanegaonkar S, Lemuth K, Vallon T, Reuss M, Armbruster W, Sprenger GA (2008). Biosynthesis of the vitamin E compound δ-tocotrienol in recombinant *Escherichia coli* cells. ChemBioChem.

[71] Kim B, Park H, Na D, Lee SY (2014). Metabolic engineering of *Escherichia coli* for the production of phenol from glucose. Biotechnol J.

[72] Magnus J. Method for producing phenol from renewable resources by fermentation. Patent WO 2014076113 A1; 2013.

[73] Miao L, Li Q, Diao A, Zhang X, Ma Y (2015). Construction of a novel phenol synthetic pathway in *Escherichia coli* through 4-hydroxybenzoate decarboxylation. Appl Microbiol Biotechnol.

[74] Thompson B, Machas M, Nielsen DR (2016). Engineering and comparison of non-natural pathways for microbial phenol production. Biotechnol Bioeng.

[75] Lin Y, Sun X, Yuan Q, Yan Y (2014). Extending shikimate pathway for the production of muconic acid and its precursor salicylic acid in *Escherichia coli*. Metab Eng.

[76] Koma D, Yamanaka H, Moriyoshi K, Ohmoto T, Sakai K (2012). Production of aromatic compounds by metabolically engineered *Escherichia coli* with an expanded shikimate pathway. Appl Environ Microbiol.

[77] Vannelli T, Qi WW, Sweigard J, Gatenby AA, Sariaslani FS (2007). Production of p-hydroxycinnamic acid from glucose in *Saccharomyces cerevisiae* and *Escherichia coli* by expression of heterologous genes from plants and fungi. Metab Eng.

[78] McKenna R, Nielsen DR (2011). Styrene biosynthesis from glucose by engineered *E. coli*. Metab Eng.

[79] Noda S, Kondo A (2017). Recent advances in microbial production of aromatic chemicals and derivatives. Trends Biotechnol.

[80] Vargas-Tah A, Martinez LM, Hernandez-Chavez G, Rocha M, Martinez A, Bolivar F, Gosset G (2015). Production of cinnamic and *p*-hydroxycinnamic acid from sugar mixtures with engineered *Escherichia coli*. Microb Cell Fact.

[81] Satoh Y, Tajima K, Munekata M, Keasling JD, Lee TS (2012). Engineering of a tyrosol-producing pathway, utilizing simple sugar and the central metabolic tyrosine, in *Escherichia coli*. J Agric Food Chem.

[82] Bloch SE, Schmidt-Dannert C (2014). Construction of a chimeric biosynthetic pathway for the de novo biosynthesis of rosmarinic acid in *Escherichia coli*. ChemBioChem.

[83] Jiang M, Zhang H (2016). Engineering the shikimate pathway for biosynthesis of molecules with pharmaceutical activities in *E. coli*. Curr Opin Biotechnol.

[84] Rodrigues AL, Trachtmann N, Becker J, Lohanatha AF, Blotenberg J, Bolten CJ, Korneli C, Lima AOD, Porto LM, Sprenger GA, Wittmann C (2013). Systems metabolic engineering of *Escherichia coli* for production of the antitumor drugs violacein and deoxyviolacein. Metab Eng.

[85] Nakagawa A, Minami H, Kim JS, Koyanagi T, Katayama T, Sato F, Kumagai H (2011). A bacterial platform for fermentative production of plant alkaloids. Nat Commun.

[86] Nakagawa A, Matsumura E, Koyanagi T, Katayama T, Kawano N, Yoshimatsu K, Yamamoto K, Kumagai H, Sato F, Minami H (2016). Total biosynthesis of opiates by stepwise fermentation using engineered *Escherichia coli*. Nat Commun.

[87] Wang J, Shen X, Rey J, Yuan Q, Yan Y (2018). Recent advances in microbial production of aromatic natural products and their derivatives. Appl Microbiol Biotechnol.

[88] Kawaguchi H, Ogino C, Kondo A (2017). Microbial conversion of biomass into bio-based polymers. Bioresour Technol.

[89] Sprenger GA, Wendisch VF (2007). Aromatic amino acids. Amino acid biosynthesis—pathways, regulation and metabolic engineering.

[90] Bailey JE (1991). Toward a science of metabolic engineering. Science.

[91] Powell KS, Cooper PD, Forneck A (2013). The biology, physiology and host-plant interactions of grape phylloxera *Daktulosphaira vitifoliae*. Behav Physiol Root Herbiv.

[92] Lutke-Eversloh T, Stephanopoulos G (2007). l-Tyrosine production by deregulated strains of *Escherichia coli*. Appl Microbiol Biotechnol.

[93] Koketsu K, Mitsuhashi S, Tabata K (2013). Identification of homophenylalanine biosynthetic genes from the cyanobacterium *Nostoc punctiforme* PCC73102 and application to its microbial production by *Escherichia coli*. Appl Environ Microbiol.

[94] Munoz AJ, Hernandez-Chavez G, de Anda R, Martinez A, Bolivar F, Gosset G (2011). Metabolic engineering of *Escherichia coli* for improving l-3,4-dihydroxyphenylalanine (l-DOPA) synthesis from glucose. J Ind Microbiol Biotechnol.

[95] Wei T, Cheng BY, Liu JZ (2016). Genome engineering *Escherichia coli* for L-DOPA overproduction from glucose. Sci Rep.

[96] Muller U, van Assema F, Gunsior M, Orf S, Kremer S, Schipper D, Wagemans A, Townsend CA, Sonke T, Bovenberg R, Wubbolts M (2006). Metabolic engineering of the *E. coli*l-phenylalanine pathway for the production of d-phenylglycine (d-Phg). Metab Eng.

[97] Meiswinkel TM, Rittmann D, Lindner SN, Wendisch VF (2013). Crude glycerol-based production of amino acids and putrescine by *Corynebacterium glutamicum*. Bioresour Technol.

[98] Escalante A, Salinas Cervantes A, Gosset G, Bolivar F (2012). Current knowledge of the *Escherichia coli* phosphoenolpyruvate-carbohydrate phosphotransferase system: peculiarities of regulation and impact on growth and product formation. Appl Microbiol Biotechnol.

[99] Herrmann KM (1995). The shikimate pathway as an entry to aromatic secondary metabolism. Plant Physiol.

[100] Andersen KB, von Meyenburg K (1980). Are growth rates of *Escherichia coli* in batch cultures limited by respiration?. J Bacteriol.

[101] Yazdani SS, Gonzalez R (2007). Anaerobic fermentation of glycerol: a path to economic viability for the biofuels industry. Curr Opin Biotechnol.

[102] Ahn JO, Lee HW, Saha R, Park MS, Jung JK, Lee DY (2008). Exploring the effects of carbon sources on the metabolic capacity for shikimic acid production in *Escherichia coli* using in silico metabolic predictions. J Microbiol Biotechnol.

[103] Salgado H, Gama-Castro S, Peralta-Gil M, Diaz-Peredo E, Sanchez-Solano F, Santos-Zavaleta A, Martinez-Flores I, Jimenez-Jacinto V, Bonavides-Martinez C, Segura-Salazar J (2006). RegulonDB (version 5.0): *Escherichia coli* K-12 transcriptional regulatory network, operon organization, and growth conditions. Nucleic Acids Res.

[104] Sambrook J, Fritsch EF, Maniatis T (2001). Molecular cloning: a laboratory manual.

