# Supplementary material for: Production of p-amino-l-phenylalanine (l-PAPA) from glycerol by metabolic grafting of Escherichia coli
Source: Microb Cell Fact. 2018 Sep 21;17:149. doi: 10.1186/s12934-018-0996-6 (PMC6148955; doi:10.1186/s12934-018-0996-6)
Supplement: Supplementary file 1 — Additional file 1. Construction and sequences of vectors [file 12934_2018_996_MOESM1_ESM.docx]

Additional data to the manuscript

Production of *p*-amino-l-phenylalanine (l-PAPA) from glycerol by metabolic grafting of *Escherichia coli*

Behrouz Mohammadi Nargesi, Natalie Trachtmann, Georg A. Sprenger, Jung-Won Youn*

Institute of Microbiology, University of Stuttgart

Allmandring 31, D-70569 Stuttgart, Germany

* corresponding author, e-mail address: jung-won.youn@imb.uni-stuttgart.de

**Construction of Vectors**

The DNA including the regions of the P*_tac_* promoter and the multiple cloning site (MCS; *Eco*RI to *Hin*dIII) was excised from vector pJF119EH (Fürste et al. 1986) by *Ssp*I digestion (643bp fragment prepared) and then ligated with the likewise digested pJem2 (Jeske & Altenbuchner 2010) vector DNA. This ligation mix was transformed into strain DH5α with selection on kanamycin resistance. From Km^R^- transformants, plasmid DNA was extracted and verified by restriction analysis and DNA - sequencing (GATC Biotech, Konstanz, Germany). The resulting plasmid DNA (pJem-P_tac_) was then digested by *Hind*III and the large linear fragment (4335 bp) was circularized by self-ligation to remove the DNA fragment containing *rha*P_BAD_–T7le-eGFP. After selection of the recombinant transformants, plasmid pJem-P*tac*-del-*Hin*dIII was isolated. pJem-P*_tac_*-del-*Hin*dIII DNA was digested with *Age*I restriction enzyme and the overhanging single-strand DNA was filled in with Klenow DNA polymerase to obtain a ‘blunt’ end. Subsequently, the plasmid was digested with *Hind*III. This pJem-P_tac_-del-*Hind*III/*Age*I-Klenow-*Hind*III(blunt end) fragment was then ligated with a *Ssp*I (blunt end)-*Hin*dIII DNA fragment containing the *rrnB*T1T2 transcription terminator (from pJF119EH) downstream of its MCS region. The resulting circularized plasmid was named pJNT522 and is depicted in Fig S1.

1. Overview of steps leading to pJNT522


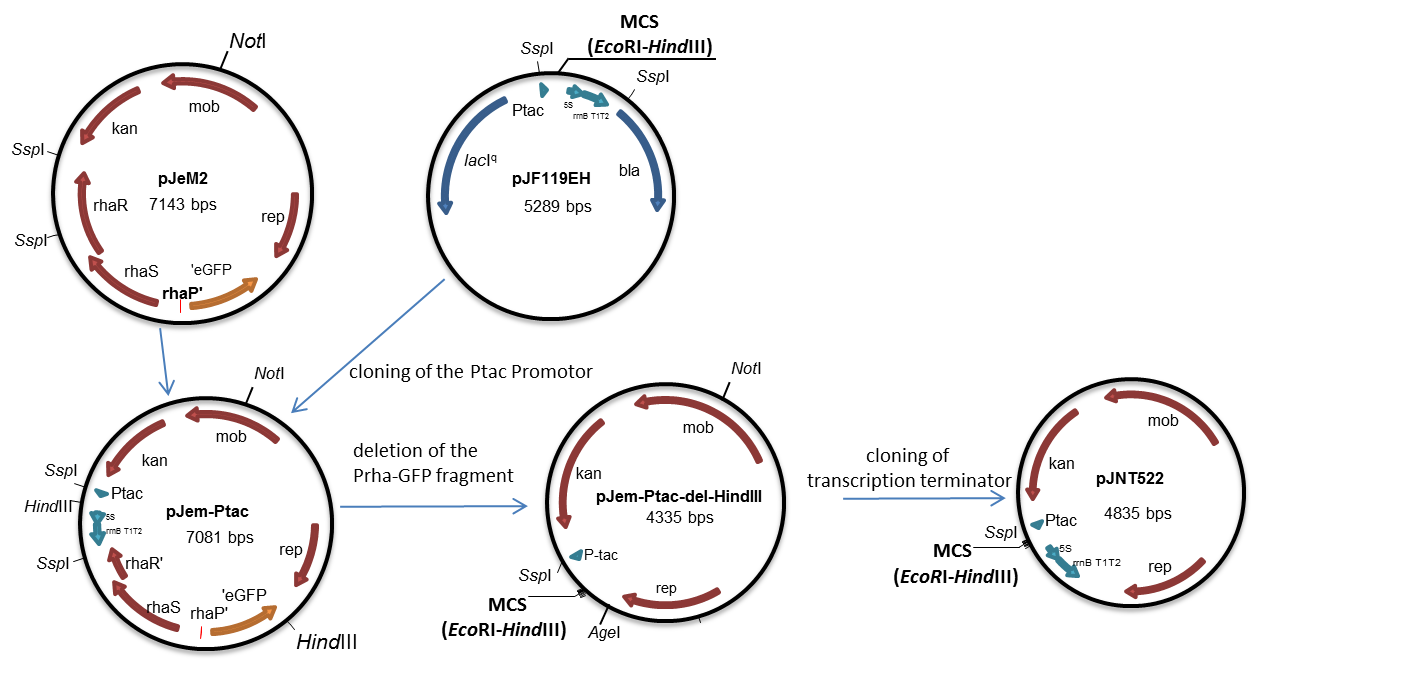


1. pJNT522

Figure S1. A) Construction of vector pJNT522 and b) map of vector pJNT522. The DNA depicted by red arrows stems from plasmid pJF119EH, the remaining part is from pJeM2.

DNA Sequence of pJNT522 vector (4835bp)

ACCTTCGGGAGCGCCTGAAGCCCGTTCTGGACGCCCTGGGGCCGTTGAATCGGGATATGCAGGCCAAGGCCGCCGCGATCATCAAGGCCGTGGGCGAAAAGCTGCTGACGGAACAGCGGGAAGTCCAGCGCCAGAAACAGGCCCAGCGCCAGCAGGAACGCGGGCGCGCACATTTCCCCGAAAAGTGCCACCTGGGATGAATGTCAGCTACTGGGCTATCTGGACAAGGGAAAACGCAAGCGCAAAGAGAAAGCAGGTAGCTTGCAGTGGGCTTACATGGCGATAGCTAGACTGGGCGGTTTTATGGACAGCAAGCGAACCGGAATTGCCAGCTGGGGCGCCCTCTGGTAAGGTTGGGAAGCCCTGCAAAGTAAACTGGATGGCTTTCTTGCCGCCAAGGATCTGATGGCGCAGGGGATCAAGATCTGATCAAGAGACAGGATGAGGATCGTTTCGCATGATTGAACAAGATGGATTGCACGCAGGTTCTCCGGCCGCTTGGGTGGAGAGGCTATTCGGCTATGACTGGGCACAACAGACAATCGGCTGCTCTGATGCCGCCGTGTTCCGGCTGTCAGCGCAGGGGCGCCCGGTTCTTTTTGTCAAGACCGACCTGTCCGGTGCCCTGAATGAACTGCAGGACGAGGCAGCGCGGCTATCGTGGCTGGCCACGACGGGCGTTCCTTGCGCAGCTGTGCTCGACGTTGTCACTGAAGCGGGAAGGGACTGGCTGCTATTGGGCGAAGTGCCGGGGCAGGATCTCCTGTCATCTCACCTTGCTCCTGCCGAGAAAGTATCCATCATGGCTGATGCAATGCGGCGGCTGCATACGCTTGATCCGGCTACCTGCCCATTCGACCACCAAGCGAAACATCGCATCGAGCGAGCACGTACTCGGATGGAAGCCGGTCTTGTCGATCAGGATGATCTGGACGAAGAGCATCAGGGGCTCGCGCCAGCCGAACTGTTCGCCAGGCTCAAGGCGCGCATGCCCGACGGCGAGGATCTCGTCGTGACCCATGGCGATGCCTGCTTGCCGAATATCATGGTGGAAAATGGCCGCTTTTCTGGATTCATCGACTGTGGCCGGCTGGGTGTGGCGGACCGCTATCAGGACATAGCGTTGGCTACCCGTGATATTGCTGAAGAGCTTGGCGGCGAATGGGCTGACCGCTTCCTCGTGCTTTACGGTATCGCCGCTCCCGATTCGCAGCGCATCGCCTTCTATCGCCTTCTTGACGAGTTCTTCTGAGCGGGACTCTGGGGTTCGAAATGACCGACCAAGCGACGCCCAACCTGCCATCACGAGATTTCGATTCCACCGCCGCCTTCTATGAAAGGTTGGGCTTCGGAATCGTTTTCCGGGACGCCGGCTGGATGATCCTCCAGCGCGGGGATCTCATGCTGGAGTTCTTCGCCCACCCCCATGGGCAAATATTCTGAAATGAGCTGTTGACAATTAATCATCGGCTCGTATAATGTGTGGAATTGTGAGCGGATAACAATTTCACACAGGAAACAGAATTCGAGCTCGGTACCCGGGGATCCTCTAGAGTCGACCTGCAGGCATGCAAGCTTGGCTGTTTTGGCGGATGAGAGAAGATTTTCAGCCTGATACAGATTAAATCAGAACGCAGAAGCGGTCTGATAAAACAGAATTTGCCTGGCGGCAGTAGCGCGGTGGTCCCACCTGACCCCATGCCGAACTCAGAAGTGAAACGCCGTAGCGCCGATGGTAGTGTGGGGTCTCCCCATGCGAGAGTAGGGAACTGCCAGGCATCAAATAAAACGAAAGGCTCAGTCGAAAGACTGGGCCTTTCGTTTTATCTGTTGTTTGTCGGTGAACGCTCTCCTGAGTAGGACAAATCCGCCGGGAGCGGATTTGAACGTTGCGAAGCAACGGCCCGGAGGGTGGCGGGCAGGACGCCCGCCATAAACTGCCAGGCATCAAATTAAGCAGAAGGCCATCCTGACGGATGGCCTTTTTGCGTTTCTACAAACTCTTTTGTTTATTTTTCTAAATACATTCAAATATGTATCCGCTCATGAGACAATAACCCTGATAAATGCTTCAATAATCCGGTTTATTGACTACCGGAAGCAGTGTGACCGTGTGCTTCTCAAATGCCTGAGGCCAGTTTGCTCAGGCTCTCCCCGTGGAGGTAATAATTGACGATATGATCATTTATTCTGCCTCCCAGAGCCTGATAAAAACGGTGAATCCGTTAGCGAGGTGCCGCCGGCTTCCATTCAGGTCGAGGTGGCCCGGCTCCATGCACCGCGACGCAACGCGGGGAGGCAGACAAGGTATAGGGCGGCGAGGCGGCTACAGCCGATAGTCTGGAACAGCGCACTTACGGGTTGCTGCGCAACCCAAGTGCTACCGGCGCGGCAGCGTGACCCGTGTCGGCGGCTCCAACGGCTCGCCATCGTCCAGAAAACACGGCTCATCGGGCATCGGCAGGCGCTGCTGCCCGCGCCGTTCCCATTCCTCCGTTTCGGTCAAGGCTGGCAGGTCTGGTTCCATGCCCGGAATGCCGGGCTGGCTGGGCGGCTCCTCGCCGGGGCCGGTCGGTAGTTGCTGCTCGCCCGGATACAGGGTCGGGATGCGGCGCAGGTCGCCATGCCCCAACAGCGATTCGTCCTGGTCGTCGTGATCAACCACCACGGCGGCACTGAACACCGACAGGCGCAACTGGTCGCGGGGCTGGCCCCACGCCACGCGGTCATTGACCACGTAGGCCGACACGGTGCCGGGGCCGTTGAGCTTCACGACGGAGATCCAGCGCTCGGCCACCAAGTCCTTGACTGCGTATTGGACCGTCCGCAAAGAACGTCCGATGAGCTTGGAAAGTGTCTTCTGGCTGACCACCACGGCGTTCTGGTGGCCCATCTGCGCCACGAGGTGATGCAGCAGCATTGCCGCCGTGGGTTTCCTCGCAATAAGCCCGGCCCACGCCTCATGCGCTTTGCGTTCCGTTTGCACCCAGTGACCGGGCTTGTTCTTGGCTTGAATGCCGATTTCTCTGGACTGCGTGGCCATGCTTATCTCCATGCGGTAGGGTGCCGCACGGTTGCGGCACCATGCGCAATCAGCTGCAACTTTTCGGCAGCGCGACAACAATTATGCGTTGCGTAAAAGTGGCAGTCAATTACAGATTTTCTTTAACCTACGCAATGAGCTATTGCGGGGGGTGCCGCAATGAGCTGTTGCGTACCCCCCTTTTTTAAGTTGTTGATTTTTAAGTCTTTCGCATTTCGCCCTATATCTAGTTCTTTGGTGCCCAAAGAAGGGCACCCCTGCGGGGTTCCCCCACGCCTTCGGCGCGGCTCCCCCTCCGGCAAAAAGTGGCCCCTCCGGGGCTTGTTGATCGACTGCGCGGCCTTCGGCCTTGCCCAAGGTGGCGCTGCCCCCTTGGAACCCCCGCACTCGCCGCCGTGAGGCTCGGGGGGCAGGCGGGCGGGCTTCGCCTTCGACTGCCCCCACTCGCATAGGCTTGGGTCGTTCCAGGCGCGTCAAGGCCAAGCCGCTGCGCGGTCGCTGCGCGAGCCTTGACCCGCCTTCCACTTGGTGTCCAACCGGCAAGCGAAGCGCGCAGGCCGCAGGCCGGAGGCTTTTCCCCAGAGAAAATTAAAAAAATTGATGGGGCAAGGCCGCAGGCCGCGCAGTTGGAGCCGGTGGGTATGTGGTCGAAGGCTGGGTAGCCGGTGGGCAATCCCTGTGGTCAAGCTCGTGGGCAGGCGCAGCCTGTCCATCAGCTTGTCCAGCAGGGTTGTCCACGGGCCGAGCGAAGCGAGCCAGCCGGTGGCCGCTCGCGGCCATCGTCCACATATCCACGGGCTGGCAAGGGAGCGCAGCGACCGCGCAGGGCGAAGCCCGGAGAGCAAGCCCGTAGGGCGCCGCAGCCGCCGTAGGCGGTCACGACTTTGCGAAGCAAAGTCTAGTGAGTATACTCAAGCATTGAGTGGCCCGCCGGAGGCACCGCCTTGCGCTGCCCCCGTCGAGCCGGTTGGACACCAAAAGGGAGGGGCAGGCATGGCGGCATACGCGATCATGCGATGCAAGAAGCTGGCGAAAATGGGCAACGTGGCGGCCAGTCTCAAGCACGCCTACCGCGAGCGCGAGACGCCCAACGCTGACGCCAGCAGGACGCCAGAGAACGAGCACTGGGCGGCCAGCAGCACCGATGAAGCGATGGGCCGACTGCGCGAGTTGCTGCCAGAGAAGCGGCGCAAGGACGCTGTGTTGGCGGTCGAGTACGTCATGACGGCCAGCCCGGAATGGTGGAAGTCGGCCAGCCAAGAACAGCAGGCGGCGTTCTTCGAGAAGGCGCACAAGTGGCTGGCGGACAAGTACGGGGCGGATCGCATCGTGACGGCCAGCATCCACCGTGACGAAACCAGCCCGCACATGACCGCGTTCGTGGTGCCGCTGACGCAGGACGGCAGGCTGTCGGCCAAGGAGTTCATCGGCAACAAAGCGCAGATGACCCGCGACCAGACCACGTTTGCGGCCGCTGTGGCCGATCTAGGGCTGCAACGGGGCATCGAGGGCAGCAAGGCACGTCACACGCGCATTCAGGCGTTCTACGAGGCCCTGGAGCGGCCACCAGTGGGCCACGTCACCATCAGCCCGCAAGCGGTCGAGCCACGCGCCTATGCACCGCAGGGATTGGCCGAAAAGCTGGGAATCTCAAAGCGCGTTGAGACGCCGGAAGCCGTGGCCGACCGGCTGACAAAAGCGGTTCGGCAGGGGTATGAGCCTGCCCTACAGGCCGCCGCAGGAGCGCGTGAGATGCGCAAGAAGGCCGATCAAGCCCAAGAGACGGCCCGAG

Figure S2. Map of vector pF109, based upon pJF119EH

DNA Sequence of pF109 vector (7553 bp)

GAATTCGAGCATAAACAGGATCGCCATCATGCAAAAAGACGCGCTGAATAACGTACATATTACCGACGAACAGGTTTTAATGACTCCGGAACAACTGAAGGCCGCTTTTCCATTGAGCCTGCAACAAGAAGCCCAGATTGCTGACTCGCGTAAAAGCATTTCAGATATTATCGCCGGGCGCGATCCTCGTCTGCTGGTAGTATGTGGTCCTTGTTCCATTCATGATCCGGAAACTGCTCTGGAATATGCTCGTCGATTTAAAGCCCTTGCCGCAGAGGTCAGCGATAGCCTCTATCTGGTAATGCGCGTCTATTTTGAAAAACCCCGTACCACTGTCGGCTGGAAAGGGTTAATTAACGATCCCCATATGGATGGCTCTTTTGATGTAGAAGCCGGGCTGCAGATCGCGCGTAAATTGCTGCTTGAGCTGGTGAATATGGGACTGCCACTGGCGACGGAAGCGTTAGATCCGAATAGCCCGCAATACCTGGGCGATCTGTTTAGCTGGTCAGCAATTGGTGCTCGTACAACGGAATCGCAAACTCACCGTGAAATGGCCTCCGGGCTTTCCATGCCGGTTGGTTTTAAAAACGGCACCGACGGCAGTCTGGCAACAGCAATTAACGCTATGCGCGCCGCCGCCCAGCCGCACCGTTTTGTTGGCATTAACCAGGCAGGGCAGGTTGCGTTGCTACAAACTCAGGGGAATCCGGACGGCCATGTGATCCTGCGCGGTGGTAAAGCGCCGAACTATAGCCCTGCGGATGTTGCGCAATGTGAAAAAGAGATGGAACAGGCGGGACTGCGCCCGTCTCTGATGGTAGATTGCAGCCACGGTAATTCCAATAAAGATTATCGCCGTCAGCCTGCGGTGGCAGAATCCGTGGTTGCTCAAATCAAAGATGGCAATCGCTCAATTATTGGTCTGATGATCGAAAGTAATATCCACGAGGGCAATCAGTCTTCCGAGCAACCGCGCAGTGAAATGAAATACGGTGTATCCGTAACCGATGCCTGCATTAGCTGGGAAATGACCGATGCCTTGCTGCGTGAAATTCATCAGGATCTGAACGGGCAGCTGACGGCTCGCGTGGCTTAAGAGGTTTATTATGGTTGCTGAATTGACCGCATTACGCGATCAAATTGATGAAGTCGATAAAGCGCTGCTGAATTTATTAGCGAAGCCCCGGGGGATCCGTAATTAAGGTGGATGTCGCGTTATGGAGAGGATTGTCGTTACTCTCGGGGAACGTAGTTACCCAATTACCATCGCATCTGGTTTGTTTAATGAACCAGCTTCATTCTTACCGCTGAAATCGGGCGAGCAGGTCATGTTGGTCACCAACGAAACCCTGGCTCCTCTGTATCTCGATAAGGTCCGCGGCGTACTTGAACAGGCGGGTGTTAACGTCGATAGCGTTATCCTCCCTGACGGCGAGCAGTATAAAAGCCTGGCTGTACTCGATACCGTCTTTACGGCGTTGTTACAAAAACCGCATGGTCGCGATACTACGCTGGTGGCGCTTGGCGGCGGCGTAGTGGGCGATCTGACCGGCTTCGCGGCGGCGAGTTATCAGCGCGGTGTCCGTTTCATTCAAGTCCCGACGACGTTACTGTCGCAGGTCGATTCCTCCGTTGGCGGCAAAACTGCGGTCAACCATCCCCTCGGTAAAAACATGATTGGCGCGTTCTACCAACCTGCTTCAGTGGTGGTGGATCTCGACTGTCTGAAAACGCTTCCCCCGCGTGAGTTAGCGTCGGGGCTGGCAGAAGTCATCAAATACGGCATTATTCTTGACGGTGCGTTTTTTAACTGGCTGGAAGAGAATCTGGATGCGTTGTTGCGTCTGGACGGTCCGGCAATGGCGTACTGTATTCGCCGTTGTTGTGAACTGAAGGCAGAAGTTGTCGCCGCCGACGAGCGCGAAACCGGGTTACGTGCTTTACTGAATCTGGGACACACCTTTGGTCATGCCATTGAAGCTGAAATGGGGTATGGCAATTGGTTACATGGTGAAGCGGTCGCTGCGGGTATGGTGATGGCGGCGCGGACGTCGGAACGTCTCGGGCAGTTTAGTTCTGCCGAAACGCAGCGTATTATAACCCTGCTCAAGCGGGCTGGGTTACCGGTCAATGGGCCGCGCGAAATGTCCGCGCAGGCGTATTTACCGCATATGCTGCGTGACAAGAAAGTCCTTGCGGGAGAGATGCGCTTAATTCTTCCGTTGGCAATTGGTAAGAGTGAAGTTCGCAGCGGCGTTTCGCACGAGCTTGTTCTTAACGCCATTGCCGATTGTCAATCAGCGTAACAACAAGAAAGCTTCTGTTTTGGCGGATGAGAGAAGATTTTCAGCCTGATACAGATTAAATCAGAACGCAGAAGCGGTCTGATAAAACAGAATTTGCCTGGCGGCAGTAGCGCGGTGGTCCCACCTGACCCCATGCCGAACTCAGAAGTGAAACGCCGTAGCGCCGATGGTAGTGTGGGGTCTCCCCATGCGAGAGTAGGGAACTGCCAGGCATCAAATAAAACGAAAGGCTCAGTCGAAAGACTGGGCCTTTCGTTTTATCTGTTGTTTGTCGGTGAACGCTCTCCTGAGTAGGACAAATCCGCCGGGAGCGGATTTGAACGTTGCGAAGCAACGGCCCGGAGGGTGGCGGGCAGGACGCCCGCCATAAACTGCCAGGCATCAAATTAAGCAGAAGGCCATCCTGACGGATGGCCTTTTTGCATGCCTACAAACTCTTTTGTTTATTTTTCTAAATACATTCAAATATGTATCCGCTCATGAGACAATAACCCTGATAAATGCTTCAATAATATTGAAAAAGGAAGAGTATGAGTATTCAACATTTCCGTGTCGCCCTTATTCCCTTTTTTGCGGCATTTTGCCTTCCTGTTTTTGCTCACCCAGAAACGCTGGTGAAAGTAAAAGATGCTGAAGATCAGTTGGGTGCACGAGTGGGTTACATCGAACTGGATCTCAACAGCGGTAAGATCCTTGAGAGTTTTCGCCCCGAAGAACGTTTTCCAATGATGAGCACTTTTAAAGTTCTGCTATGTGGCGCGGTATTATCCCGTGTTGACGCCGGGCAAGAGCAACTCGGTCGCCGCATACACTATTCTCAGAATGACTTGGTTGAGTACTCACCAGTCACAGAAAAGCATCTTACGGATGGCATGACAGTAAGAGAATTATGCAGTGCTGCCATAACCATGAGTGATAACACTGCGGCCAACTTACTTCTGACAACGATCGGAGGACCGAAGGAGCTAACCGCTTTTTTGCACAACATGGGGGATCATGTAACTCGCCTTGATCGTTGGGAACCGGAGCTGAATGAAGCCATACCAAACGACGAGCGTGACACCACGATGCCTGTAGCAATGGCAACAACGTTGCGCAAACTATTAACTGGCGAACTACTTACTCTAGCTTCCCGGCAACAATTAATAGACTGGATGGAGGCGGATAAAGTTGCAGGACCACTTCTGCGCTCGGCCCTTCCGGCTGGCTGGTTTATTGCTGATAAATCTGGAGCCGGTGAGCGTGGGTCTCGCGGTATCATTGCAGCACTGGGGCCAGATGGTAAGCCCTCCCGTATCGTAGTTATCTACACGACGGGGAGTCAGGCAACTATGGATGAACGAAATAGACAGATCGCTGAGATAGGTGCCTCACTGATTAAGCATTGGTAACTGTCAGACCAAGTTTACTCATATATACTTTAGATTGATTTAAAACTTCATTTTTAATTTAAAAGGATCTAGGTGAAGATCCTTTTTGATAATCTCATGACCAAAATCCCTTAACGTGAGTTTTCGTTCCACTGAGCGTCAGACCCCGTAGAAAAGATCAAAGGATCTTCTTGAGATCCTTTTTTTCTGCGCGTAATCTGCTGCTTGCAAACAAAAAAACCACCGCTACCAGCGGTGGTTTGTTTGCCGGATCAAGAGCTACCAACTCTTTTTCCGAAGGTAACTGGCTTCAGCAGAGCGCAGATACCAAATACTGTCCTTCTAGTGTAGCCGTAGTTAGGCCACCACTTCAAGAACTCTGTAGCACCGCCTACATACCTCGCTCTGCTAATCCTGTTACCAGTGGCTGCTGCCAGTGGCGATAAGTCGTGTCTTACCGGGTTGGACTCAAGACGATAGTTACCGGATAAGGCGCAGCGGTCGGGCTGAACGGGGGGTTCGTGCACACAGCCCAGCTTGGAGCGAACGACCTACACCGAACTGAGATACCTACAGCGTGAGCTATGAGAAAGCGCCACGCTTCCCGAAGGGAGAAAGGCGGACAGGTATCCGGTAAGCGGCAGGGTCGGAACAGGAGAGCGCACGAGGGAGCTTCCAGGGGGAAACGCCTGGTATCTTTATAGTCCTGTCGGGTTTCGCCACCTCTGACTTGAGCGTCGATTTTTGTGATGCTCGTCAGGGGGGCGGAGCCTATGGAAAAACGCCAGCAACGCGGCCTTTTTACGGTTCCTGGCCTTTTGCTGGCCTTTTGCTCACATGTTCTTTCCTGCGTTATCCCCTGATTCTGTGGATAACCGTATTACCGCCTTTGAGTGAGCTGATACCGCTCGCCGCAGCCGAACGACCGAGCGCAGCGAGTCAGTGAGCGAGGAAGCGGAAGAGCGCCTGATGCGGTATTTTCTCCTTACGCATCTGTGCGGTATTTCACACCGCATATGGTGCACTCTCAGTACAATCTGCTCTGATGCCGCATAGTTAAGCCAGTATACACTCCGCTATCGCTACGTGACTGGGTCATGGCTGCGCCCCGACACCCGCCAACACCCGCTGACGCGCCCTGACGGGCTTGTCTGCTCCCGGCATCCGCTTACAGACAAGCTGTGACCGTCTCCGGGAGCTGCATGTGTCAGAGGTTTTCACCGTCATCACCGAAACGCGCGAGGCAGCTGCGGTAAAGCTCATCAGCGTGGTCGTGAAGCGATTCACAGATGTCTGCCTGTTCATCCGCGTCCAGCTCGTTGAGTTTCTCCAGAAGCGTTAATGTCTGGCTTCTGATAAAGCGGGCCATGTTAAGGGCGGTTTTTTCCTGTTTGGTCACTTGATGCCTCCGTGTAAGGGGGAATTTCTGTTCATGGGGGTAATGATACCGATGAAACGAGAGAGGATGCTCACGATACGGGTTACTGATGATGAACATGCCCGGTTACTGGAACGTTGTGAGGGTAAACAACTGGCGGTATGGATGCGGCGGGACCAGAGAAAAATCACTCAGGGTCAATGCCAGCGCTTCGTTAATACAGATGTAGGTGTTCCACAGGGTAGCCAGCAGCATCCTGCGATGCAGATCCGGAACATAATGGTGCAGGGCGCTGACTTCCGCGTTTCCAGACTTTACGAAACACGGAAACCGAAGACCATTCATGTTGTTGCTCAGGTCGCAGACGTTTTGCAGCAGCAGTCGCTTCACGTTCGCTCGCGTATCGGTGATTCATTCTGCTAACCAGTAAGGCAACCCCGCCAGCCTAGCCGGGTCCTCAACGACAGGAGCACGATCATGCGCACCCGTGGCCAGGACCCAACGCTGCCCGAGATGCGCCGCGTGCGGCTGCTGGAGATGGCGGACGCGATGGATATGTTCTGCCAAGGGTTGGTTTGCGCATTCACAGTTCTCCGCAAGAATTGATTGGCTCCAATTCTTGGAGTGGTGAATCCGTTAGCGAGGTGCCGCCGGCTTCCATTCAGGTCGAGGTGGCCCGGCTCCATGCACCGCGACGCAACGCGGGGAGGCAGACAAGGTATAGGGCGGCGCCTACAATCCATGCCAACCCGTTCCATGTGCTCGCCGAGGCGGCATAAATCGCCGTGACGATCAGCGGTCCAGTGATCGAAGTTAGGCTGGTAAGAGCCGCGAGCGATCCTTGAAGCTGTCCCTGATGGTCGTCATCTACCTGCCTGGACAGCATGGCCTGCAACGCGGGCATCCCGATGCCGCCGGAAGCGAGAAGAATCATAATGGGGAAGGCCATCCAGCCTCGCGTCGCGAACGCCAGCAAGACGTAGCCCAGCGCGTCGGCCAGCTTGCAATTCGCGCTAACTTACATTAATTGCGTTGCGCTCACTGCCCGCTTTCCAGTCGGGAAACCTGTCGTGCCAGCTGCATTAATGAATCGGCCAACGCGCGGGGAGAGGCGGTTTGCGTATTGGGCGCCAGGGTGGTTTTTCTTTTCACCAGTGAGACGGGCAACAGCTGATTGCCCTTCACCGCCTGGCCCTGAGAGAGTTGCAGCAAGCGGTCCACGTGGTTTGCCCCAGCAGGCGAAAATCCTGTTTGATGGTGGTTAACGGCGGGATATAACATGAGCTGTCTTCGGTATCGTCGTATCCCACTACCGAGATATCCGCACCAACGCGCAGCCCGGACTCGGTAATGGCGCGCATTGCGCCCAGCGCCATCTGATCGTTGGCAACCAGCATCGCAGTGGGAACGATGCCCTCATTCAGCATTTGCATGGTTTGTTGAAAACCGGACATGGCACTCCAGTCGCCTTCCCGTTCCGCTATCGGCTGAATTTGATTGCGAGTGAGATATTTATGCCAGCCAGCCAGACGCAGACGCGCCGAGACAGAACTTAATGGGCCCGCTAACAGCGCGATTTGCTGGTGACCCAATGCGACCAGATGCTCCACGCCCAGTCGCGTACCGTCTTCATGGGAGAAAATAATACTGTTGATGGGTGTCTGGTCAGAGACATCAAGAAATAACGCCGGAACATTAGTGCAGGCAGCTTCCACAGCAATGGCATCCTGGTCATCCAGCGGATAGTTAATGATCAGCCCACTGACGCGTTGCGCGAGAAGATTGTGCACCGCCGCTTTACAGGCTTCGACGCCGCTTCGTTCTACCATCGACACCACCACGCTGGCACCCAGTTGATCGGCGCGAGATTTAATCGCCGCGACAATTTGCGACGGCGCGTGCAGGGCCAGACTGGAGGTGGCAACGCCAATCAGCAACGACTGTTTGCCCGCCAGTTGTTGTGCCACGCGGTTGGGAATGTAATTCAGCTCCGCCATCGCCGCTTCCACTTTTTCCCGCGTTTTCGCAGAAACGTGGCTGGCCTGGTTCACCACGCGGGAAACGGTCTGATAAGAGACACCGGCATACTCTGCGACATCGTATAACGTTACTGGTTTCACATTCACCACCCTGAATTGACTCTCTTCCGGGCGCTATCATGCCATACCGCGAAAGGTTTTGCACCATTCGATGGTGTCAACGTAAATGCCGCTTCGCCTTCGCGCGCGAATTGCAAGCTGATCCGGGCTTATCGACTGCACGGTGCACCAATGCTTCTGGCGTCAGGCAGCCATCGGAAGCTGTGGTATGGCTGTGCAGGTCGTAAATCACTGCATAATTCGTGTCGCTCAAGGCGCACTCCCGTTCTGGATAATGTTTTTTGCGCCGACATCATAACGGTTCTGGCAAATATTCTGAAATGAGCTGTTGACAATTAATCATCGGCTCGTATAATGTGTGGAATTGTGAGCGGATAACAATTTCACACAGGAAACA

Subcloning of *aroFBL* genes:

The *E. coli* *aroFB* genes were cut out from plasmid pF109 plasmid (see Fig. S2) with *Eco*RI and *Hin*dIII and ligated to a likewise cut pJNT522 vector. The *aroL* gene was excised as a small *Hin*dIII fragment from plasmid pF112 plasmid (see Fig. S3) with subsequent cloning into the pJNT-*aroFB* plasmid via *Hin*dIII restriction sites. The direction of the cloned gene was tested by restriction analysis and subsequent sequencing of resulting plasmid.

Figure S3 Map of vector pF-112, based upon pJF119EH.

Sequence of vector pF-112 (8114 base pairs)

GAATTCGAGCATAAACAGGATCGCCATCATGCAAAAAGACGCGCTGAATAACGTACATATTACCGACGAACAGGTTTTAATGACTCCGGAACAACTGAAGGCCGCTTTTCCATTGAGCCTGCAACAAGAAGCCCAGATTGCTGACTCGCGTAAAAGCATTTCAGATATTATCGCCGGGCGCGATCCTCGTCTGCTGGTAGTATGTGGTCCTTGTTCCATTCATGATCCGGAAACTGCTCTGGAATATGCTCGTCGATTTAAAGCCCTTGCCGCAGAGGTCAGCGATAGCCTCTATCTGGTAATGCGCGTCTATTTTGAAAAACCCCGTACCACTGTCGGCTGGAAAGGGTTAATTAACGATCCCCATATGGATGGCTCTTTTGATGTAGAAGCCGGGCTGCAGATCGCGCGTAAATTGCTGCTTGAGCTGGTGAATATGGGACTGCCACTGGCGACGGAAGCGTTAGATCCGAATAGCCCGCAATACCTGGGCGATCTGTTTAGCTGGTCAGCAATTGGTGCTCGTACAACGGAATCGCAAACTCACCGTGAAATGGCCTCCGGGCTTTCCATGCCGGTTGGTTTTAAAAACGGCACCGACGGCAGTCTGGCAACAGCAATTAACGCTATGCGCGCCGCCGCCCAGCCGCACCGTTTTGTTGGCATTAACCAGGCAGGGCAGGTTGCGTTGCTACAAACTCAGGGGAATCCGGACGGCCATGTGATCCTGCGCGGTGGTAAAGCGCCGAACTATAGCCCTGCGGATGTTGCGCAATGTGAAAAAGAGATGGAACAGGCGGGACTGCGCCCGTCTCTGATGGTAGATTGCAGCCACGGTAATTCCAATAAAGATTATCGCCGTCAGCCTGCGGTGGCAGAATCCGTGGTTGCTCAAATCAAAGATGGCAATCGCTCAATTATTGGTCTGATGATCGAAAGTAATATCCACGAGGGCAATCAGTCTTCCGAGCAACCGCGCAGTGAAATGAAATACGGTGTATCCGTAACCGATGCCTGCATTAGCTGGGAAATGACCGATGCCTTGCTGCGTGAAATTCATCAGGATCTGAACGGGCAGCTGACGGCTCGCGTGGCTTAAGAGGTTTATTATGGTTGCTGAATTGACCGCATTACGCGATCAAATTGATGAAGTCGATAAAGCGCTGCTGAATTTATTAGCGAAGCCCCGGGGGATCCGTAATTAAGGTGGATGTCGCGTTATGGAGAGGATTGTCGTTACTCTCGGGGAACGTAGTTACCCAATTACCATCGCATCTGGTTTGTTTAATGAACCAGCTTCATTCTTACCGCTGAAATCGGGCGAGCAGGTCATGTTGGTCACCAACGAAACCCTGGCTCCTCTGTATCTCGATAAGGTCCGCGGCGTACTTGAACAGGCGGGTGTTAACGTCGATAGCGTTATCCTCCCTGACGGCGAGCAGTATAAAAGCCTGGCTGTACTCGATACCGTCTTTACGGCGTTGTTACAAAAACCGCATGGTCGCGATACTACGCTGGTGGCGCTTGGCGGCGGCGTAGTGGGCGATCTGACCGGCTTCGCGGCGGCGAGTTATCAGCGCGGTGTCCGTTTCATTCAAGTCCCGACGACGTTACTGTCGCAGGTCGATTCCTCCGTTGGCGGCAAAACTGCGGTCAACCATCCCCTCGGTAAAAACATGATTGGCGCGTTCTACCAACCTGCTTCAGTGGTGGTGGATCTCGACTGTCTGAAAACGCTTCCCCCGCGTGAGTTAGCGTCGGGGCTGGCAGAAGTCATCAAATACGGCATTATTCTTGACGGTGCGTTTTTTAACTGGCTGGAAGAGAATCTGGATGCGTTGTTGCGTCTGGACGGTCCGGCAATGGCGTACTGTATTCGCCGTTGTTGTGAACTGAAGGCAGAAGTTGTCGCCGCCGACGAGCGCGAAACCGGGTTACGTGCTTTACTGAATCTGGGACACACCTTTGGTCATGCCATTGAAGCTGAAATGGGGTATGGCAATTGGTTACATGGTGAAGCGGTCGCTGCGGGTATGGTGATGGCGGCGCGGACGTCGGAACGTCTCGGGCAGTTTAGTTCTGCCGAAACGCAGCGTATTATAACCCTGCTCAAGCGGGCTGGGTTACCGGTCAATGGGCCGCGCGAAATGTCCGCGCAGGCGTATTTACCGCATATGCTGCGTGACAAGAAAGTCCTTGCGGGAGAGATGCGCTTAATTCTTCCGTTGGCAATTGGTAAGAGTGAAGTTCGCAGCGGCGTTTCGCACGAGCTTGTTCTTAACGCCATTGCCGATTGTCAATCAGCGTAACAACAAGAAAGCTTCTATTGGGGAAAACCCACGATGACACAACCTCTTTTTCTGATCGGGCCTCGGGGCTGTGGTAAAACAACGGTCGGAATGGCCCTTGCCGATTCGCTTAACCGTCGGTTTGTCGATACCGATCAGTGGTTGCAATCACAGCTCAATATGACGGTCGCGGAGATCGTCGAAAGGGAAGAGTGGGCGGGATTTCGCGCCAGAGAAACGGCGGCGCTGGAAGCGGTAACTGCGCCATCCACCGTTATCGCTACAGGCGGCGGCATTATTCTGACGGAATTTAATCGTCACTTCATGCAAAATAACGGGATCGTGGTTTATTTGTGTGCGCCAGTATCAGTCCTGGTTAACCGACTGCAAGCTGCACCGGAAGAAGATTTACGGCCAACCTTAACGGGAAAACCGCTGAGCGAAGAAGTTCAGGAAGTGCTGGAAGAACGCGATGCGCTATATCGCGAAGTTGCGCATATTATCATCGACGCAACAAACGAACCCAGCCAGGTGATTTCTGAAATTCGCAGCGCCCTGGCACAGACGATCAATTGTTGATTTTCGAGCGCAAGCTTCTGTTTTGGCGGATGAGAGAAGATTTTCAGCCTGATACAGATTAAATCAGAACGCAGAAGCGGTCTGATAAAACAGAATTTGCCTGGCGGCAGTAGCGCGGTGGTCCCACCTGACCCCATGCCGAACTCAGAAGTGAAACGCCGTAGCGCCGATGGTAGTGTGGGGTCTCCCCATGCGAGAGTAGGGAACTGCCAGGCATCAAATAAAACGAAAGGCTCAGTCGAAAGACTGGGCCTTTCGTTTTATCTGTTGTTTGTCGGTGAACGCTCTCCTGAGTAGGACAAATCCGCCGGGAGCGGATTTGAACGTTGCGAAGCAACGGCCCGGAGGGTGGCGGGCAGGACGCCCGCCATAAACTGCCAGGCATCAAATTAAGCAGAAGGCCATCCTGACGGATGGCCTTTTTGCATGCCTACAAACTCTTTTGTTTATTTTTCTAAATACATTCAAATATGTATCCGCTCATGAGACAATAACCCTGATAAATGCTTCAATAATATTGAAAAAGGAAGAGTATGAGTATTCAACATTTCCGTGTCGCCCTTATTCCCTTTTTTGCGGCATTTTGCCTTCCTGTTTTTGCTCACCCAGAAACGCTGGTGAAAGTAAAAGATGCTGAAGATCAGTTGGGTGCACGAGTGGGTTACATCGAACTGGATCTCAACAGCGGTAAGATCCTTGAGAGTTTTCGCCCCGAAGAACGTTTTCCAATGATGAGCACTTTTAAAGTTCTGCTATGTGGCGCGGTATTATCCCGTGTTGACGCCGGGCAAGAGCAACTCGGTCGCCGCATACACTATTCTCAGAATGACTTGGTTGAGTACTCACCAGTCACAGAAAAGCATCTTACGGATGGCATGACAGTAAGAGAATTATGCAGTGCTGCCATAACCATGAGTGATAACACTGCGGCCAACTTACTTCTGACAACGATCGGAGGACCGAAGGAGCTAACCGCTTTTTTGCACAACATGGGGGATCATGTAACTCGCCTTGATCGTTGGGAACCGGAGCTGAATGAAGCCATACCAAACGACGAGCGTGACACCACGATGCCTGTAGCAATGGCAACAACGTTGCGCAAACTATTAACTGGCGAACTACTTACTCTAGCTTCCCGGCAACAATTAATAGACTGGATGGAGGCGGATAAAGTTGCAGGACCACTTCTGCGCTCGGCCCTTCCGGCTGGCTGGTTTATTGCTGATAAATCTGGAGCCGGTGAGCGTGGGTCTCGCGGTATCATTGCAGCACTGGGGCCAGATGGTAAGCCCTCCCGTATCGTAGTTATCTACACGACGGGGAGTCAGGCAACTATGGATGAACGAAATAGACAGATCGCTGAGATAGGTGCCTCACTGATTAAGCATTGGTAACTGTCAGACCAAGTTTACTCATATATACTTTAGATTGATTTAAAACTTCATTTTTAATTTAAAAGGATCTAGGTGAAGATCCTTTTTGATAATCTCATGACCAAAATCCCTTAACGTGAGTTTTCGTTCCACTGAGCGTCAGACCCCGTAGAAAAGATCAAAGGATCTTCTTGAGATCCTTTTTTTCTGCGCGTAATCTGCTGCTTGCAAACAAAAAAACCACCGCTACCAGCGGTGGTTTGTTTGCCGGATCAAGAGCTACCAACTCTTTTTCCGAAGGTAACTGGCTTCAGCAGAGCGCAGATACCAAATACTGTCCTTCTAGTGTAGCCGTAGTTAGGCCACCACTTCAAGAACTCTGTAGCACCGCCTACATACCTCGCTCTGCTAATCCTGTTACCAGTGGCTGCTGCCAGTGGCGATAAGTCGTGTCTTACCGGGTTGGACTCAAGACGATAGTTACCGGATAAGGCGCAGCGGTCGGGCTGAACGGGGGGTTCGTGCACACAGCCCAGCTTGGAGCGAACGACCTACACCGAACTGAGATACCTACAGCGTGAGCTATGAGAAAGCGCCACGCTTCCCGAAGGGAGAAAGGCGGACAGGTATCCGGTAAGCGGCAGGGTCGGAACAGGAGAGCGCACGAGGGAGCTTCCAGGGGGAAACGCCTGGTATCTTTATAGTCCTGTCGGGTTTCGCCACCTCTGACTTGAGCGTCGATTTTTGTGATGCTCGTCAGGGGGGCGGAGCCTATGGAAAAACGCCAGCAACGCGGCCTTTTTACGGTTCCTGGCCTTTTGCTGGCCTTTTGCTCACATGTTCTTTCCTGCGTTATCCCCTGATTCTGTGGATAACCGTATTACCGCCTTTGAGTGAGCTGATACCGCTCGCCGCAGCCGAACGACCGAGCGCAGCGAGTCAGTGAGCGAGGAAGCGGAAGAGCGCCTGATGCGGTATTTTCTCCTTACGCATCTGTGCGGTATTTCACACCGCATATGGTGCACTCTCAGTACAATCTGCTCTGATGCCGCATAGTTAAGCCAGTATACACTCCGCTATCGCTACGTGACTGGGTCATGGCTGCGCCCCGACACCCGCCAACACCCGCTGACGCGCCCTGACGGGCTTGTCTGCTCCCGGCATCCGCTTACAGACAAGCTGTGACCGTCTCCGGGAGCTGCATGTGTCAGAGGTTTTCACCGTCATCACCGAAACGCGCGAGGCAGCTGCGGTAAAGCTCATCAGCGTGGTCGTGAAGCGATTCACAGATGTCTGCCTGTTCATCCGCGTCCAGCTCGTTGAGTTTCTCCAGAAGCGTTAATGTCTGGCTTCTGATAAAGCGGGCCATGTTAAGGGCGGTTTTTTCCTGTTTGGTCACTTGATGCCTCCGTGTAAGGGGGAATTTCTGTTCATGGGGGTAATGATACCGATGAAACGAGAGAGGATGCTCACGATACGGGTTACTGATGATGAACATGCCCGGTTACTGGAACGTTGTGAGGGTAAACAACTGGCGGTATGGATGCGGCGGGACCAGAGAAAAATCACTCAGGGTCAATGCCAGCGCTTCGTTAATACAGATGTAGGTGTTCCACAGGGTAGCCAGCAGCATCCTGCGATGCAGATCCGGAACATAATGGTGCAGGGCGCTGACTTCCGCGTTTCCAGACTTTACGAAACACGGAAACCGAAGACCATTCATGTTGTTGCTCAGGTCGCAGACGTTTTGCAGCAGCAGTCGCTTCACGTTCGCTCGCGTATCGGTGATTCATTCTGCTAACCAGTAAGGCAACCCCGCCAGCCTAGCCGGGTCCTCAACGACAGGAGCACGATCATGCGCACCCGTGGCCAGGACCCAACGCTGCCCGAGATGCGCCGCGTGCGGCTGCTGGAGATGGCGGACGCGATGGATATGTTCTGCCAAGGGTTGGTTTGCGCATTCACAGTTCTCCGCAAGAATTGATTGGCTCCAATTCTTGGAGTGGTGAATCCGTTAGCGAGGTGCCGCCGGCTTCCATTCAGGTCGAGGTGGCCCGGCTCCATGCACCGCGACGCAACGCGGGGAGGCAGACAAGGTATAGGGCGGCGCCTACAATCCATGCCAACCCGTTCCATGTGCTCGCCGAGGCGGCATAAATCGCCGTGACGATCAGCGGTCCAGTGATCGAAGTTAGGCTGGTAAGAGCCGCGAGCGATCCTTGAAGCTGTCCCTGATGGTCGTCATCTACCTGCCTGGACAGCATGGCCTGCAACGCGGGCATCCCGATGCCGCCGGAAGCGAGAAGAATCATAATGGGGAAGGCCATCCAGCCTCGCGTCGCGAACGCCAGCAAGACGTAGCCCAGCGCGTCGGCCAGCTTGCAATTCGCGCTAACTTACATTAATTGCGTTGCGCTCACTGCCCGCTTTCCAGTCGGGAAACCTGTCGTGCCAGCTGCATTAATGAATCGGCCAACGCGCGGGGAGAGGCGGTTTGCGTATTGGGCGCCAGGGTGGTTTTTCTTTTCACCAGTGAGACGGGCAACAGCTGATTGCCCTTCACCGCCTGGCCCTGAGAGAGTTGCAGCAAGCGGTCCACGTGGTTTGCCCCAGCAGGCGAAAATCCTGTTTGATGGTGGTTAACGGCGGGATATAACATGAGCTGTCTTCGGTATCGTCGTATCCCACTACCGAGATATCCGCACCAACGCGCAGCCCGGACTCGGTAATGGCGCGCATTGCGCCCAGCGCCATCTGATCGTTGGCAACCAGCATCGCAGTGGGAACGATGCCCTCATTCAGCATTTGCATGGTTTGTTGAAAACCGGACATGGCACTCCAGTCGCCTTCCCGTTCCGCTATCGGCTGAATTTGATTGCGAGTGAGATATTTATGCCAGCCAGCCAGACGCAGACGCGCCGAGACAGAACTTAATGGGCCCGCTAACAGCGCGATTTGCTGGTGACCCAATGCGACCAGATGCTCCACGCCCAGTCGCGTACCGTCTTCATGGGAGAAAATAATACTGTTGATGGGTGTCTGGTCAGAGACATCAAGAAATAACGCCGGAACATTAGTGCAGGCAGCTTCCACAGCAATGGCATCCTGGTCATCCAGCGGATAGTTAATGATCAGCCCACTGACGCGTTGCGCGAGAAGATTGTGCACCGCCGCTTTACAGGCTTCGACGCCGCTTCGTTCTACCATCGACACCACCACGCTGGCACCCAGTTGATCGGCGCGAGATTTAATCGCCGCGACAATTTGCGACGGCGCGTGCAGGGCCAGACTGGAGGTGGCAACGCCAATCAGCAACGACTGTTTGCCCGCCAGTTGTTGTGCCACGCGGTTGGGAATGTAATTCAGCTCCGCCATCGCCGCTTCCACTTTTTCCCGCGTTTTCGCAGAAACGTGGCTGGCCTGGTTCACCACGCGGGAAACGGTCTGATAAGAGACACCGGCATACTCTGCGACATCGTATAACGTTACTGGTTTCACATTCACCACCCTGAATTGACTCTCTTCCGGGCGCTATCATGCCATACCGCGAAAGGTTTTGCACCATTCGATGGTGTCAACGTAAATGCCGCTTCGCCTTCGCGCGCGAATTGCAAGCTGATCCGGGCTTATCGACTGCACGGTGCACCAATGCTTCTGGCGTCAGGCAGCCATCGGAAGCTGTGGTATGGCTGTGCAGGTCGTAAATCACTGCATAATTCGTGTCGCTCAAGGCGCACTCCCGTTCTGGATAATGTTTTTTGCGCCGACATCATAACGGTTCTGGCAAATATTCTGAAATGAGCTGTTGACAATTAATCATCGGCTCGTATAATGTGTGGAATTGTGAGCGGATAACAATTTCACACAGGAAACA

Plasmid pC53BC

Figure S4 Plasmid map of pC53BC, based upon vector pJF119EH

DNA sequence of pC53BC (8503 base pairs)

GAATTCGAGCTCGGTACCCGGGGATCTGTGGTTTTGTCAGAGGATGTCATGCGCGTTTTAATTATTGATAATTATGATTCTTTCACGTTTAATCTCGCCACCTATGTGGAAGAGGTTACGGGTCAGGCACCTGTGGTGGTGCCTAATGATCAAGAAATAGATGAGATGCTTTTCGACGCCGTCATCCTCTCACCTGGCCCGGGCCACGCCGGCGTTGCGGCTGATTTTGGTATCTGTGCAGGCGTCATTGAGCGTGCACGCGTTCCGATTTTGGGTGTGTGTTTAGGCCACCAGGGCATTGCGTTGGCCTATGGCGGTGATGTTGATTTGGCGCCCAGGCCGGTCCACGGTGAGGTTTCGCAGATCACCCATGATGGTTCAGGTTTATTTGCAGGCATCCCTGAAACGTTTGAGGCGGTGCGTTATCACTCGATGGTGGCAACCCGCTTGCCGGAGTCATTGAAAGCTACAGCTACCAGCGATGATGGTTTGATCATGGCATTGGCACATGAAGTGCTTCCGCAGTGGGGTGTGCAATTTCATCCGGAATCTATTGGTGGACAATTCGGCCATCAGATCATTAAGAACTTCCTTAATTTAGCGCGCACATATCGCTGGCAACTCACGGAGAAAACTATTCCGCTCAGCGTTGATTCAGCAGCGGTTTTTGAAACATTCTTTGCCCATTCCTCCCATGCTTTTTGGCTCGATGATGCCCAAGGAACCAGCTATCTTGGTGATGCCAGCGGTCCTCTCGCACGCACAAAAACCCATAATGTCGGCGAGGGGGATTTCTTCACCTGGCTAAAGGAGGATCTCGCCGCCAACTCAGTTGCGCCCGGTCAAGGTTTTCGTCTTGGCTGGGTTGGTTACGTTGGTTATGAGCTTAAAGCGGAAGCTGGCGCACGGGCTGCGCACACTTCGAGTCTTCCGGATGCGCACCTCATTTTTGCCGATCGCGCCATCGCAGTGGAATCGGATCAGGTTCGGTTGCTGGCGTTGGGGGAGCAGGACGAGTGGTTTGAAGAAACCATCAAGAAGCTGCATAATCTTGTCGCCCCGCGGATACCTGCGTCCGGACACCTCGCTTTGCAGGTTCGAGATTCCAAAGATGAGTATCTCGACAAAATTCGCAGAGCCCAGGAGCTGATTACTCGCGGCGAATCGTATGAAATCTGCCTGACCACAAAACTTCAGGGCACCACTGATGTGGCCCCTCTGGCTGCCTATCTAGCACTGCGTGGGGCCAATCCCACCGCATATGGTGCGTATCTTCAGCTGGGGGATACCTCTATTTTGAGTTCCTCGCCGGAGCGGTTCATCACCATTGATTCGGCAGGGTATGTGGAATCAAAGCCCATTAAAGGCACCAGGCCGCGTGGGCGAACAGCGCAAGAAGACCAAGAAATCATTGCTGAGCTGCGCAGTAATCCTAAAGATCGTGCAGAAAACTTGATGATCGTGGATTTGGTCCGCAACGACTTAGCCCGCGGCGCTTTGCCCACCACAGTTAAAACATCCAAGCTTTTCGACGTCGAAACCTACGCCACAGTCCACCAACTTGTCAGCACCGTCTCTGCAGAGTTGGGGCCACGCAGTCCGATTGAGTGCGTGCGCGCAGCATTCCCCGGTGGTTCGATGACTGGTGCCCCAAAGCTGCGCACCATGGAGATCATCGATGAGCTGGAGGCAGCTCCTCGCGGTATTTACTCAGGTGGCTTGGGATATTTTTCCCTCGACGGCGCAGTTGATCTCTCCATGGTGATCAGAACTCTCGTCATCCAGAACAATCACGTGGAGTACGGAGTGGGCGGTGCACTTCTTGCTCTGTCTGATCCGGAGGCTGAGTGGGAGGAAATCCGCGTTAAATCACGGCCTCTGCTGAATTTGTTTGGGGTTGAATTCCCATGACGTACGGATCTGAAGGAGATATCATATGACCGAACAGAATGAACTGCAGCGTCTGCGTGCAGAACTGGATGCACTGGATGGCACCCTGCTGGATACCGTTCGTCGTCGTATTGATCTGGGTGTTCGTATTGCACGTTATAAAAGCCGTCATGGTGTTCCGATGATGCAGCCTGGTCGTGTTAGCCTGGTTAAAGATCGTGCAGCACGTTATGCAGCAGATCATGGTCTGGATGAAAGTTTTCTGGTGAATCTGTATGATGTGATCATCACCGAAATGTGTCGTGTTGAGGATCTGGTTATGAGCCGTGAAAGCCTGACCGCAGAAGATCGTCGTTAAGTCGACGAAGGAGATATACCATGAGCGGTTTTCCGCGTAGCGTTGTTGTTGGTGGTAGCGGTGCAGTGGGTGGTATGTTTGCAGGTCTGCTGCGTGAAGCAGGTAGCCGTACCCTGGTTGTTGATCTGGTTCCGCCTCCGGGTCGTCCGGATGCATGTCTGGTTGGTGATGTTACCGCACCGGGTCCGGAACTGGCAGCAGCACTGCGTGATGCCGATCTGGTGCTGCTGGCCGTTCATGAAGATGTTGCACTGAAAGCAGTTGCACCGGTTACCCGTCTGATGCGTCCGGGTGCACTGCTGGCAGATACCCTGAGCGTTCGTACCGGTATGGCAGCAGAGCTGGCAGCACATGCACCGGGTGTTCAGCATGTTGGTCTGAATCCGATGTTTGCACCGGCAGCAGGTATGACAGGCCGTCCGGTTGCAGCAGTTGTTACCCGTGATGGTCCTGGTGTGACCGCACTGCTGCGTCTGGTTGAAGGTGGTGGTGGTCGTCCTGTTCGTCTGACAGCCGAAGAACATGATCGTACCACCGCAGCAACCCAGGCACTGACCCATGCAGTTCTGCTGAGCTTTGGTCTGGCACTGGCACGTCTGGGTGTGGATGTTCGTGCACTGGCAGCCACCGCACCGCCTCCGCATCAAGTACTGCTGGCCCTGCTGGCACGTGTTCTGGGTGGTAGTCCGGAAGTTTATGGTGATATTCAGCGTAGCAATCCGCGTGCAGCAAGCGCACGTCGTGCCCTGGCTGAAGCCCTGCGTAGCTTTGCAGCACTGGTTGGAGATGATCCTGATCGTGCCGATGCACCAGGTCGCGCAGATGCCCCTGGTCATCCGGGTGGTTGTGATGGTGCAGGTAATCTGGATGGTGTTTTTGGTGAACTGCGTCGCCTGATGGGTCCTGAGCTGGCTGCAGGCCAGGATCATTGTCAAGAACTGTTTCGTACCCTGCATCGTACCGATGATGAAGGTGAAAAAGATCGCTAAGGATCCTCTAGAGTCGACCTGCAGGCATGCAAGCTTCTGTTTTGGCGGATGAGAGAAGATTTTCAGCCTGATACAGATTAAATCAGAACGCAGAAGCGGTCTGATAAAACAGAATTTGCCTGGCGGCAGTAGCGCGGTGGTCCCACCTGACCCCATGCCGAACTCAGAAGTGAAACGCCGTAGCGCCGATGGTAGTGTGGGGTCTCCCCATGCGAGAGTAGGGAACTGCCAGGCATCAAATAAAACGAAAGGCTCAGTCGAAAGACTGGGCCTTTCGTTTTATCTGTTGTTTGTCGGTGAACGCTCTCCTGAGTAGGACAAATCCGCCGGGAGCGGATTTGAACGTTGCGAAGCAACGGCCCGGAGGGTGGCGGGCAGGACGCCCGCCATAAACTGCCAGGCATCAAATTAAGCAGAAGGCCATCCTGACGGATGGCCTTTTTGCGTTTCTACAAACTCTTTTGTTTATTTTTCTAAATACATTCAAATATGTATCCGCTCATGAGACAATAACCCTGATAAATGCTTCAATAATATTGAAAAAGGAAGAGTATGAGTATTCAACATTTCCGTGTCGCCCTTATTCCCTTTTTTGCGGCATTTTGCCTTCCTGTTTTTGCTCACCCAGAAACGCTGGTGAAAGTAAAAGATGCTGAAGATCAGTTGGGTGCACGAGTGGGTTACATCGAACTGGATCTCAACAGCGGTAAGATCCTTGAGAGTTTTCGCCCCGAAGAACGTTTTCCAATGATGAGCACTTTTAAAGTTCTGCTATGTGGCGCGGTATTATCCCGTGTTGACGCCGGGCAAGAGCAACTCGGTCGCCGCATACACTATTCTCAGAATGACTTGGTTGAGTACTCACCAGTCACAGAAAAGCATCTTACGGATGGCATGACAGTAAGAGAATTATGCAGTGCTGCCATAACCATGAGTGATAACACTGCGGCCAACTTACTTCTGACAACGATCGGAGGACCGAAGGAGCTAACCGCTTTTTTGCACAACATGGGGGATCATGTAACTCGCCTTGATCGTTGGGAACCGGAGCTGAATGAAGCCATACCAAACGACGAGCGTGACACCACGATGCCTGTAGCAATGGCAACAACGTTGCGCAAACTATTAACTGGCGAACTACTTACTCTAGCTTCCCGGCAACAATTAATAGACTGGATGGAGGCGGATAAAGTTGCAGGACCACTTCTGCGCTCGGCCCTTCCGGCTGGCTGGTTTATTGCTGATAAATCTGGAGCCGGTGAGCGTGGGTCTCGCGGTATCATTGCAGCACTGGGGCCAGATGGTAAGCCCTCCCGTATCGTAGTTATCTACACGACGGGGAGTCAGGCAACTATGGATGAACGAAATAGACAGATCGCTGAGATAGGTGCCTCACTGATTAAGCATTGGTAACTGTCAGACCAAGTTTACTCATATATACTTTAGATTGATTTAAAACTTCATTTTTAATTTAAAAGGATCTAGGTGAAGATCCTTTTTGATAATCTCATGACCAAAATCCCTTAACGTGAGTTTTCGTTCCACTGAGCGTCAGACCCCGTAGAAAAGATCAAAGGATCTTCTTGAGATCCTTTTTTTCTGCGCGTAATCTGCTGCTTGCAAACAAAAAAACCACCGCTACCAGCGGTGGTTTGTTTGCCGGATCAAGAGCTACCAACTCTTTTTCCGAAGGTAACTGGCTTCAGCAGAGCGCAGATACCAAATACTGTCCTTCTAGTGTAGCCGTAGTTAGGCCACCACTTCAAGAACTCTGTAGCACCGCCTACATACCTCGCTCTGCTAATCCTGTTACCAGTGGCTGCTGCCAGTGGCGATAAGTCGTGTCTTACCGGGTTGGACTCAAGACGATAGTTACCGGATAAGGCGCAGCGGTCGGGCTGAACGGGGGGTTCGTGCACACAGCCCAGCTTGGAGCGAACGACCTACACCGAACTGAGATACCTACAGCGTGAGCTATGAGAAAGCGCCACGCTTCCCGAAGGGAGAAAGGCGGACAGGTATCCGGTAAGCGGCAGGGTCGGAACAGGAGAGCGCACGAGGGAGCTTCCAGGGGGAAACGCCTGGTATCTTTATAGTCCTGTCGGGTTTCGCCACCTCTGACTTGAGCGTCGATTTTTGTGATGCTCGTCAGGGGGGCGGAGCCTATGGAAAAACGCCAGCAACGCGGCCTTTTTACGGTTCCTGGCCTTTTGCTGGCCTTTTGCTCACATGTTCTTTCCTGCGTTATCCCCTGATTCTGTGGATAACCGTATTACCGCCTTTGAGTGAGCTGATACCGCTCGCCGCAGCCGAACGACCGAGCGCAGCGAGTCAGTGAGCGAGGAAGCGGAAGAGCGCCTGATGCGGTATTTTCTCCTTACGCATCTGTGCGGTATTTCACACCGCATATGGTGCACTCTCAGTACAATCTGCTCTGATGCCGCATAGTTAAGCCAGTATACACTCCGCTATCGCTACGTGACTGGGTCATGGCTGCGCCCCGACACCCGCCAACACCCGCTGACGCGCCCTGACGGGCTTGTCTGCTCCCGGCATCCGCTTACAGACAAGCTGTGACCGTCTCCGGGAGCTGCATGTGTCAGAGGTTTTCACCGTCATCACCGAAACGCGCGAGGCAGCTGCGGTAAAGCTCATCAGCGTGGTCGTGAAGCGATTCACAGATGTCTGCCTGTTCATCCGCGTCCAGCTCGTTGAGTTTCTCCAGAAGCGTTAATGTCTGGCTTCTGATAAAGCGGGCCATGTTAAGGGCGGTTTTTTCCTGTTTGGTCACTTGATGCCTCCGTGTAAGGGGGAATTTCTGTTCATGGGGGTAATGATACCGATGAAACGAGAGAGGATGCTCACGATACGGGTTACTGATGATGAACATGCCCGGTTACTGGAACGTTGTGAGGGTAAACAACTGGCGGTATGGATGCGGCGGGACCAGAGAAAAATCACTCAGGGTCAATGCCAGCGCTTCGTTAATACAGATGTAGGTGTTCCACAGGGTAGCCAGCAGCATCCTGCGATGCAGATCCGGAACATAATGGTGCAGGGCGCTGACTTCCGCGTTTCCAGACTTTACGAAACACGGAAACCGAAGACCATTCATGTTGTTGCTCAGGTCGCAGACGTTTTGCAGCAGCAGTCGCTTCACGTTCGCTCGCGTATCGGTGATTCATTCTGCTAACCAGTAAGGCAACCCCGCCAGCCTAGCCGGGTCCTCAACGACAGGAGCACGATCATGCGCACCCGTGGCCAGGACCCAACGCTGCCCGAGATGCGCCGCGTGCGGCTGCTGGAGATGGCGGACGCGATGGATATGTTCTGCCAAGGGTTGGTTTGCGCATTCACAGTTCTCCGCAAGAATTGATTGGCTCCAATTCTTGGAGTGGTGAATCCGTTAGCGAGGTGCCGCCGGCTTCCATTCAGGTCGAGGTGGCCCGGCTCCATGCACCGCGACGCAACGCGGGGAGGCAGACAAGGTATAGGGCGGCGCCTACAATCCATGCCAACCCGTTCCATGTGCTCGCCGAGGCGGCATAAATCGCCGTGACGATCAGCGGTCCAGTGATCGAAGTTAGGCTGGTAAGAGCCGCGAGCGATCCTTGAAGCTGTCCCTGATGGTCGTCATCTACCTGCCTGGACAGCATGGCCTGCAACGCGGGCATCCCGATGCCGCCGGAAGCGAGAAGAATCATAATGGGGAAGGCCATCCAGCCTCGCGTCGCGAACGCCAGCAAGACGTAGCCCAGCGCGTCGGCCAGCTTGCAATTCGCGCTAACTTACATTAATTGCGTTGCGCTCACTGCCCGCTTTCCAGTCGGGAAACCTGTCGTGCCAGCTGCATTAATGAATCGGCCAACGCGCGGGGAGAGGCGGTTTGCGTATTGGGCGCCAGGGTGGTTTTTCTTTTCACCAGTGAGACGGGCAACAGCTGATTGCCCTTCACCGCCTGGCCCTGAGAGAGTTGCAGCAAGCGGTCCACGTGGTTTGCCCCAGCAGGCGAAAATCCTGTTTGATGGTGGTTAACGGCGGGATATAACATGAGCTGTCTTCGGTATCGTCGTATCCCACTACCGAGATATCCGCACCAACGCGCAGCCCGGACTCGGTAATGGCGCGCATTGCGCCCAGCGCCATCTGATCGTTGGCAACCAGCATCGCAGTGGGAACGATGCCCTCATTCAGCATTTGCATGGTTTGTTGAAAACCGGACATGGCACTCCAGTCGCCTTCCCGTTCCGCTATCGGCTGAATTTGATTGCGAGTGAGATATTTATGCCAGCCAGCCAGACGCAGACGCGCCGAGACAGAACTTAATGGGCCCGCTAACAGCGCGATTTGCTGGTGACCCAATGCGACCAGATGCTCCACGCCCAGTCGCGTACCGTCTTCATGGGAGAAAATAATACTGTTGATGGGTGTCTGGTCAGAGACATCAAGAAATAACGCCGGAACATTAGTGCAGGCAGCTTCCACAGCAATGGCATCCTGGTCATCCAGCGGATAGTTAATGATCAGCCCACTGACGCGTTGCGCGAGAAGATTGTGCACCGCCGCTTTACAGGCTTCGACGCCGCTTCGTTCTACCATCGACACCACCACGCTGGCACCCAGTTGATCGGCGCGAGATTTAATCGCCGCGACAATTTGCGACGGCGCGTGCAGGGCCAGACTGGAGGTGGCAACGCCAATCAGCAACGACTGTTTGCCCGCCAGTTGTTGTGCCACGCGGTTGGGAATGTAATTCAGCTCCGCCATCGCCGCTTCCACTTTTTCCCGCGTTTTCGCAGAAACGTGGCTGGCCTGGTTCACCACGCGGGAAACGGTCTGATAAGAGACACCGGCATACTCTGCGACATCGTATAACGTTACTGGTTTCACATTCACCACCCTGAATTGACTCTCTTCCGGGCGCTATCATGCCATACCGCGAAAGGTTTTGCACCATTCGATGGTGTCAACGTAAATGCCGCTTCGCCTTCGCGCGCGAATTGCAAGCTGATCCGGGCTTATCGACTGCACGGTGCACCAATGCTTCTGGCGTCAGGCAGCCATCGGAAGCTGTGGTATGGCTGTGCAGGTCGTAAATCACTGCATAATTCGTGTCGCTCAAGGCGCACTCCCGTTCTGGATAATGTTTTTTGCGCCGACATCATAACGGTTCTGGCAAATATTCTGAAATGAGCTGTTGACAATTAATCATCGGCTCGTATAATGTGTGGAATTGTGAGCGGATAACAATTTCACACAGGAAACA
