# Supplementary material for: Production of p-amino-l-phenylalanine (l-PAPA) from glycerol by metabolic grafting of Escherichia coli
Source: Microb Cell Fact. 2018 Sep 21;17:149. doi: 10.1186/s12934-018-0996-6 (PMC6148955; doi:10.1186/s12934-018-0996-6)
Supplement: Supplementary file 2 — Additional file 2. Comparison of mass spectra of commercially available L-PAPA (A) with fermentatively produced L-PAPA from E. coli FUS4.7R/pC53BC/pJNTaroFBL (B). The mass spectra were recorded with an Agilent 6130 mass spectrometer system with electron spray ionization in positive mode (Agilent Technologies; Germany). In both measurements the L-PAPA+H+ mass of 181 was detected. [file 12934_2018_996_MOESM2_ESM.pdf]

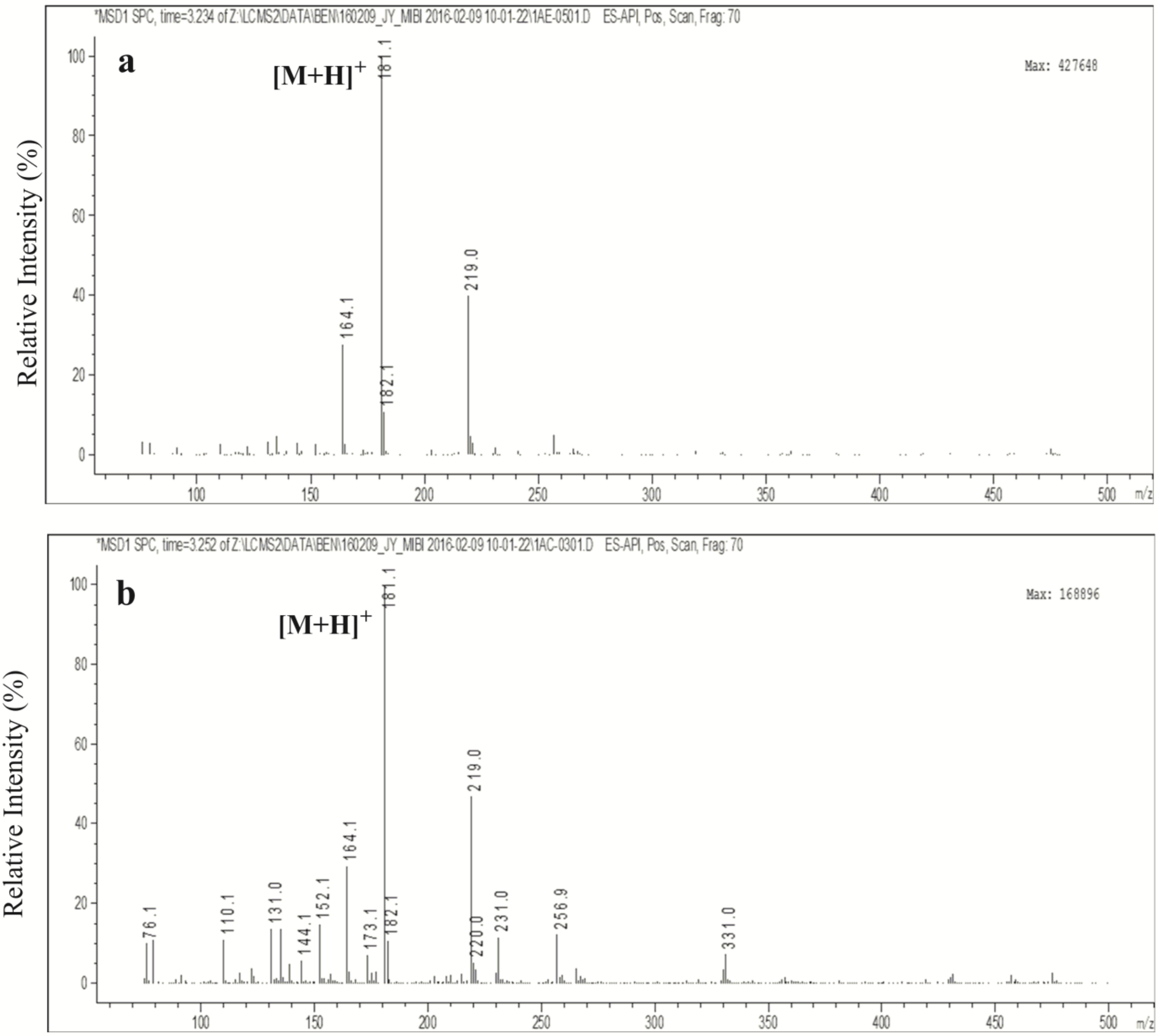

Additional file 2: Comparison of mass spectra of commercially available L-PAPA (A) with fermentatively produced L-PAPA from *E. coli* FUS4.7R /pC53BC /pJNT*aroFBL* (B). The mass spectra were recorded with an Agilent 6130 mass spectrometer system with electron spray ionization in positive mode (Agilent Technologies; Germany). In both measurements the L-PAPA+H^+ mass of 181 was detected.
